# The role of chicken management practices in children’s exposure to environmental contamination: a mixed-methods analysis

**DOI:** 10.1186/s12889-021-11025-y

**Published:** 2021-06-08

**Authors:** Simone Passarelli, Ramya Ambikapathi, Nilupa S. Gunaratna, Isabel Madzorera, Chelsey R. Canavan, Ramadhani Abdallah Noor, Dagmawit Tewahido, Yemane Berhane, Simbarashe Sibanda, Lindiwe Majele Sibanda, Tshilidzi Madzivhandila, Bertha L. M. Munthali, Margaret McConnell, Christopher Sudfeld, Kirsten Davison, Wafaie Fawzi

**Affiliations:** 1 Department of Nutrition, Harvard T. H. Chan School of Public Health, 665 Huntington Avenue, Boston, MA 02115 USA; 2 Department of Public Health, Purdue University, West Lafayette, USA; 3 Department of Global Health and Population, Harvard T. H. Chan School of Public Health, Boston, USA; 4 Addis Continental Institute of Public Health, Addis Ababa, Ethiopia; 5 Food, Agriculture and Natural Resources Policy Analysis Network, Pretoria, South Africa; 6 Boston College, School of Social Work, Chestnut Hill, USA; 7 Department of Epidemiology, Harvard T. H. Chan School of Public Health, Boston, USA

**Keywords:** Agriculture, Water, sanitation, and hygiene, Ethiopia, Child health, Nutrition

## Abstract

**Background:**

Household chicken production presents an opportunity to promote child nutrition, but the benefits might be offset by increased environmental contamination. Using household surveys, direct observations, and in-depth interviews with woman caregivers, we sought to describe the relationship between chicken management practices and household exposure to environmental contamination, and assess barriers to adopting improved husbandry practices.

**Methods:**

First, we analyzed baseline data from 973 households raising chickens in the two interventions arms from the Agriculture-to-Nutrition (ATONU) study in Ethiopia to assess the relationship between animal management practices and environmental exposures. Second, we conducted six-hour direct observations of children’s environmental exposures in 18 households. Among these households, we analyzed in-depth interviews with child caregivers.

**Results:**

Quantitative analyses showed that households raised approximately 11 chickens, had animal feces visible on the property 67% of the time, and children’s hands were visibly dirty 38% of the time. Households with more chickens had lower exposure to animal feces. Having a chicken coop increased the risk of observing animal feces on the property by 30%, but among those with a coop, having an enclosed coop reduced that risk by 83%. Coops that were enclosed, had fencing, and were located further from homes were associated with a reduced risk of observing animal feces and an increased likelihood of children having clean hands. Direct observations showed that chicken coops were often poorly designed or not used. On average, 3 to 5 chickens were inside homes at a time, and livestock and domestic animals were frequently inside of houses and interacting with young children. In-depth interviews revealed that protection of animals, maintenance of household cleanliness and health, type of chicken (local versus improved) and resource constraints influenced management decisions.

**Conclusions:**

Improvements in chicken management practices could mitigate the exposure of household members to environmental contamination. Our findings highlight the need for training and resources to promote safe animal husbandry practices and optimal child health in nutrition-sensitive livestock projects.

**Trial registration:**

Clinical trials number: NCT03152227; Retrospectively registered at ClinicalTrials.gov on May 12, 2012.

**Supplementary Information:**

The online version contains supplementary material available at 10.1186/s12889-021-11025-y.

## Background

In Ethiopia, approximately 40% of children under five suffer from chronic undernutrition [1]. To address this issue among rural populations that face poor physical and financial access to healthy diets, agricultural approaches have been promoted as one potential way to support nutrition [2, 3]. Specifically, the production of animal source foods (ASF) has been recognized as a promising enterprise that can potentially empower women—who are often responsible for raising small animals—while also diversifying household diets and incomes, and increasing access to micronutrient-rich foods [4–6].

However, there are concerns that small-scale animal production may increase a household’s exposure to contamination, especially in areas with suboptimal water, sanitation, and hygiene conditions [7, 8]. Contamination of houses and yards with animal feces can lead to the spread of pathogens in water, food, and hands, resulting in the ingestion of fecal matter that leads to infection and, consequently, threats to the health and nutrition of infants and young children [7, 9]. The main pathways through which exposure to animal contamination can harm child nutritional status are through the transmission of zoonotic pathogens that cause diarrhea, through parasitic infections such as worms, and through a subclinical condition known as environmental enteric dysfunction (EED), which triggers low-level inflammation and poor nutrient absorption [7, 9, 10]. Thus, increased ownership of animals may be associated with increased exposure to fecal matter [11] which has been associated with a higher prevalence of child growth faltering [10–13].

Recent research has attempted to address these concerns of animal ownership and contamination by conducting observational, experimental, and qualitative studies. Some methods to explore these relationships have included “spot checks” of cleanliness around a household [11]; direct observations of infants and young children to document instances of exposure to contamination [13, 14]; direct measurement of pathogens and their transmission to humans, animals, and objects [14–17]; measurement of biomarkers of EED [18]; randomized trials to limit exposure to contamination [19–21]; description of the factors predicting chicken corralling practices by households [22]; qualitative research to understand perceptions surrounding the adoption of animal corrals [23, 24]; and a combination of these methods [14, 25]. However, we are not aware of any studies that quantitatively describe the chicken management practices used by households and then assess them qualitatively for ground-truthing purposes, especially in the context of Ethiopia. Moreover, our mixed methods include the use of in-depth interviews, which provide a detailed perspective on decision-making processes and justification for the use of husbandry practices by households.

Due to the recent push towards nutrition-sensitive livestock [3], it is important to evaluate whether rural, resource-constrained households can safely integrate animals into their environments. Thus, as part of the baseline household survey of the Agriculture-to-Nutrition (ATONU) trial, an intervention designed to increase the production and consumption of chickens in rural Ethiopia, we collected a number of indicators describing chicken management practices and the households’ interaction with animals. Based on previous research showing a high degree of exposure of young children to environmental health risks [11, 14], additional data triangulation through direct observations and in-depth interviews was necessary in order provide a more validated view of chicken management practices in this context.

The aims of this paper were to:

1. Show the relationship between chicken management practices and distal measures of sanitation, using quantitative baseline data from the ATONU trial;
2. Describe the pathways of chicken-child interactions using qualitative direct observation data collected during the midline of the ATONU trial; and.
3. Investigate the beliefs and barriers underlying the adoption of different chicken management practices by households through qualitative in-depth interviews with woman caregivers of young children.

## Methods

### Theoretical approach

The theory used to inform our methodology was based on the conceptual framework from Passarelli et al., 2020 [26]. This framework describes the hypothesized pathways between chicken production and child nutrition and health based on evidence from two main areas: 1) the agriculture-nutrition pathways identified by Harris and Herforth [27], which include income, diet, and women’s empowerment, and 2) the pathways through which exposure to fecal contamination could adversely affect nutrition and health [7, 11, 12, 15].

We undertook a mixed-methods approach for several reasons. First, the indicators from the household survey used to describe chicken management are crude when analyzed on their own, and thus provide an incomplete picture of environmental contamination. Ground-truthing through direct observations can help to better describe the implications of management practices for household members, especially young children who are the most vulnerable to infection. Moreover, while previous work has described the dangers of exposure to environmental contamination from animals, qualitative evidence is needed to contextualize these findings. In order to provide culturally- appropriate, evidence-based solutions for future programs, we must understand the barriers farmers face that prevent them from adopting safer practices. We hypothesized that practices limiting the contact between animals and people would be associated with reductions in markers of contamination, but that resource, knowledge, and cultural barriers would limit their adoption.

**Fig. 1:**
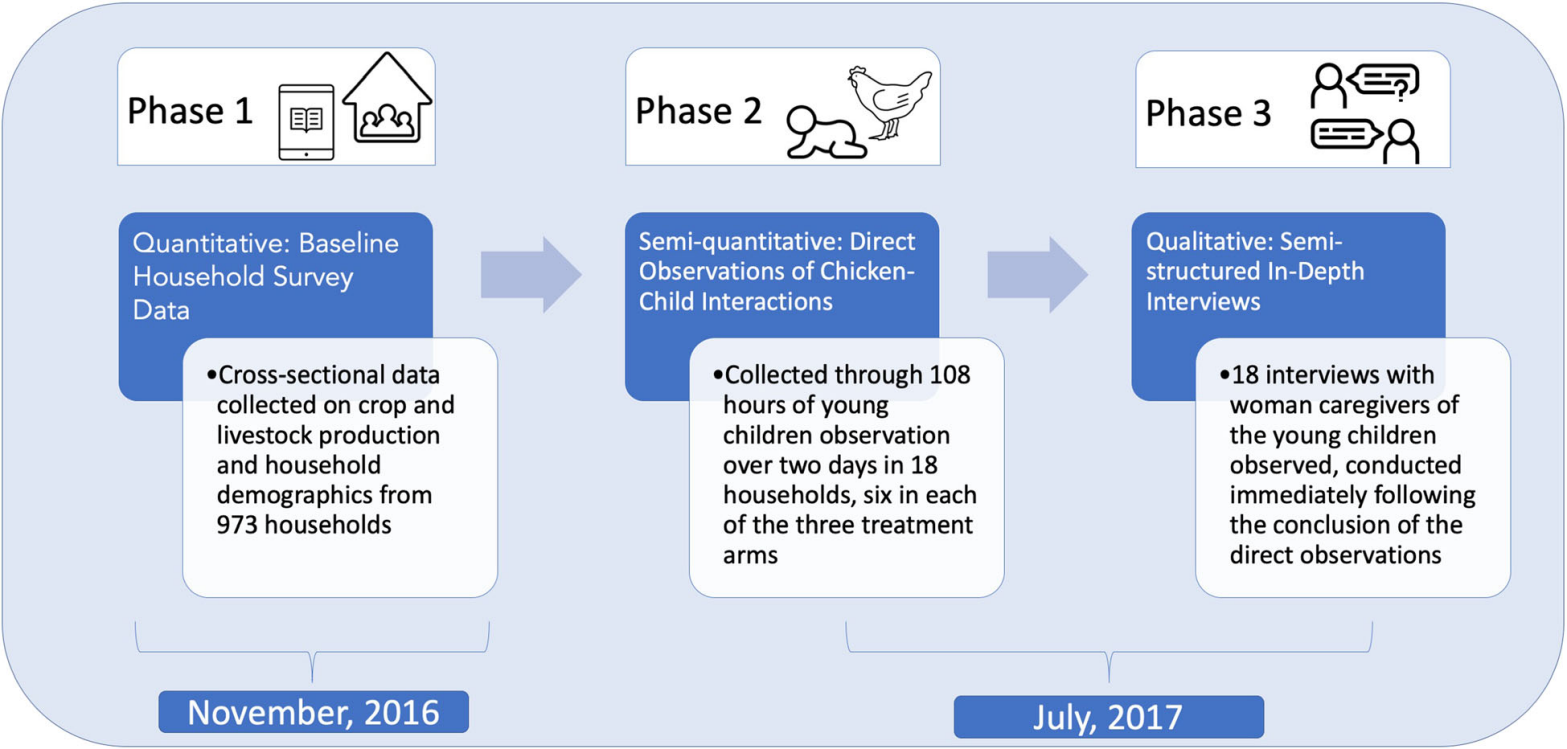
Description of the mixed methods used in this study

To address the three stated study objectives, we used an explanatory sequential mixed-methods design with three phases (see Fig. 1). Exploratory sequential mixed-methods research involves first collecting quantitative data and then using them to inform the collection of in-depth qualitative data [28]. In the first phase, quantitative data were collected using household surveys with participants in the ATONU study to assess current chicken management practices and the degree of exposure to contamination among households. During the second phase, semi-quantitative direct observation data were collected through two-day direct observations of households with young children (0–36 months of age at baseline). In the third phase, semi-structured in-depth interviews were conducted with the primary woman caregivers of those same children. The data from these three phases were integrated at both the methods level, by sampling households for the qualitative data collection that participated in the household survey, and at the interpretation and reporting level, through a contiguous discussion in the results section, followed by a weaving approach in the discussion section, based on the integration principles suggested by Fetters et al. [29].

### Quantitative methods: phase 1

#### Study design

This study was conducted as a substudy of Agriculture-to-Nutrition (ATONU) (clinicaltrials.gov identifier # NCT03152227), a cluster-randomized trial designed to evaluate the impact of integrating nutrition-sensitive behavioral change communication, in the context of increased household production of chickens and eggs, on the diets of women and children.

In short, both intervention arms, ACGG and ACGG+ATONU, participated in the African Chicken Genetic Gains intervention, which included the receipt of 25 high-yielding chicks from five hybrid breeds and technical assistance on chicken production and management [26]. Additionally, only the ACGG+ATONU arm received a behavior change communication (BCC) intervention on nutrition, child feeding practices, WASH behaviors, women’s empowerment, budgeting and savings, home gardening practices, and vegetable seed. The control arm received no intervention other than data collection. As part of participation in the ACGG intervention, households were advised to build a chicken coop using their own materials; to use a semi-scavenging system where chickens forage freely for several hours, twice per day to graze on available food in the environment; and to provide birds with supplemental feed. However, actual chicken husbandry practices were implemented by farmers according to their individual preferences and means.

#### Setting

The larger ATONU trial was implemented in four regions of Ethiopia; for the purposes of this sub-study, we limited the quantitative analyses to the Amhara and Oromia regions in order for the quantitative analyses to parallel the regions studied under the qualitative phases. Within each region, 20 districts were selected based on pre-determined criteria for having a suitable agroecology for chicken production. Villages were randomly selected from within districts and randomly assigned to one of three treatment arms: 1) African Chicken Genetic Gains (ACGG), 2) ACGG+ATONU, or 3) control. This analysis includes 15 villages in Amhara and 18 in Oromia, allocated in equal proportions to the three treatment arms.

#### Participants

The inclusion criteria for the ATONU study consisted of having produced chickens for at least 2 years, having fewer than 50 birds, having at least one woman of reproductive age (18–49 years at enrollment), and providing informed consent. From these eligible households in a village, 35 were randomly selected to participate in the intervention. One index child was enrolled in the study if there was a child aged 0–36 months living in the household at baseline.

#### Variables

The primary exposures of interest were the chicken management practices used by households. These were measured using the following five variables: 1) household had a chicken coop (binary; yes/no); 2) number of chickens owned (categorical; 1–3 chickens, 4–9 chickens, or 10+ chickens); 3) whether chickens slept in the house the previous night (binary; yes/no); and among the 814 households that reported having a chicken coop, the following questions were asked about the coop: 4) type of chicken coop (categorical; open housing, enclosed housing, fencing, or other); and 5) distance of chicken coop to house (categorical; 0 meters/attached to or inside of house, 1–3 meters, or 4+ meters). The primary outcomes of interest were potential sources of environmental contamination to which young children might be exposed, as measured by 1) whether there were feces visible on the property, and 2) whether the children’s hands appeared to be clean. We examined the relationship between the five measures of chicken husbandry practices and two binary measures of exposure to contamination based on associations shown between these factors and health outcomes in the literature [7, 9, 11, 17, 30].

#### Data sources and measurement

The household survey collected data on crop and livestock production, household water, sanitation, and hygiene (WASH) characteristics, demographics, physical household characteristics, income, expenditures, child health, women’s empowerment, and nutrition. All quantitative analyses (excluding the semi-quantitative direct observation data) presented in this paper are from the baseline household survey; the questionnaire was developed for the ACGG+ATONU evaluation and has been previously published in the baseline report [31]. Exposure variables related to characteristics of the chicken coop were asked of the primary woman decisionmaker in the household, since women are generally responsible for raising chickens in this context [32, 33]. The outcome variables for the presence of animal feces and whether the child’s hands were clean were based on enumerator observation. Standards were developed during the survey training and piloting until these observations were similar across enumerators.

#### Sample size

Of the 2117 households in the ATONU sample, 262 households without chickens at baseline were excluded from the analysis. We further restricted the sample to the 973 households in the Oromia (*n* = 500) and Amhara (*n* = 473) regions in order for the quantitative results to be representative of the qualitative sample. For analyses that focused on the cleanliness of the index child’s hands, the sample was limited to the households that enrolled an index child between the ages of 0–36 months at baseline (*n* = 431).

#### Statistical methods

Confounder variables were the same for all adjusted models and included the following baseline demographic characteristics: having improved water (yes/no, based on WHO/UNICEF definition [34]), having improved sanitation (yes/no, based on WHO/UNICEF definition [34]), number of household members (1–4; 5–7; 8+), region (Amhara or Oromia), agroecological zone (lowland, midland, highland), wealth quintile (based on the quintiles of the first principal component of asset ownership), whether the household was woman-headed, education level of the woman of reproductive age (none; primary 1; primary 2; secondary 1 or higher; other such as adult literacy/religious school), and age of the woman respondent (15–30; 31–38; 39+). We also calculated tertiles of a score of women’s decision making in chicken production, based on the share of the following six activities for which women reported participating in at least some decisions, relative to few or no decisions: chicken production, chicken inputs, use of eggs, marketing of eggs, slaughter of chickens, and marketing of chickens. We also controlled for the number of other (non-poultry) livestock owned (0–3; 4–7; 8+) in order to adjust for differences in environmental exposures that were due to ownership of other animals. For all variables, missing observations were entered as a separate category (not shown in tables) for 39 values for women’s education, 13 for improved water, and 4 for improved sanitation.

For regressions analyzing the relationships of chicken management practices with exposure to contamination, we conducted both adjusted and unadjusted regressions using log binomial models to estimate risk ratios and 95% confidence intervals. Poisson distributions were used to overcome issues of non-convergence. For all regressions presented in this paper, robust confidence intervals were clustered at the level of the village, which was the unit of enrollment of eligible households, and bootstrapped with 500 iterations [35].

#### Bias

We controlled for all known confounders that we hypothesized could bias the exposure-outcome relationship. One potential source of bias is that households were told that they would be participating in the ACGG program before the baseline survey took place. Consequently, they may have started implementing improved chicken management practices that would be captured in our baseline data. However, because the purpose of the quantitative analysis was to describe how different management practices related to measures of exposure to environmental contamination, we did not attempt to analyze differences across treatments arms; rather, we adjusted for treatment arm as a confounder in the models.

### Phases 2 and 3: qualitative methods

#### Study design

To address our second research question, a complementary qualitative study was conducted in six villages during the midline of the intervention’s implementation and evaluation in July of 2017. This research included direct household observations and in-depth interviews with caregivers of children who were 0–36 months of age at baseline. These villages were selected from Amhara and Oromia regions, which were chosen based on perceived representativeness (being Ethiopia’s two largest regions by population) while meeting resource constraints for transportation. In order to observe differences by treatment group, we randomly selected one ATONU village, one ACGG+ATONU village, and one control village from each of the two regions. Three households were randomly chosen from each village, for a total of 18 households.

#### Direct observation data collection

For the direct observations, enumerators observed the 18 households for 3 h per day over 2 days (6 h per household), totaling 108 h. The semi-quantitative data collection instrument was modified from the tool used in formative research for the SHINE trial [14] with permission of the authors and is available in Supplement 2. The instrument included a combination of pre-coded and open-ended questions to allow enumerators flexibility in describing what they observed, while also ensuring comparability of indicators across sites. The protocol included data on the number of times livestock and children occupied the same space; where the children were sitting or playing; animal housing; cleanliness of the child, mother and compound; the child’s tactile and oral contact with objects; and domestic water, sanitation, and hygiene practices and conditions. These data were collected through both hourly spot checks (at 0, 1, 2, and 3 h during each of the 2 days), and tallies of all behaviors observed over the 3 h on both days.

#### Direct observation data analysis

The direct observation data were analyzed to assess the prevalence and frequency of environmental exposures and protective behaviors observed across the 6 h of observations. We analyzed the observation data from eight instances of observation—at the beginning and end of each hour of observation (i.e. at hours zero, one, two and three)—to assess the proximity of the index child to animals at that given moment. For these observation data, the mean for each household was taken across the 2 days, and then the mean across the 18 households was calculated. We also assessed the average number of animals observed inside the house over the course of the 6 h of observation at the eight observation time points. This average was calculated based on the number of each animal type (including bovines, sheep, cats, dogs, and chickens) seen across each household at each of the time points, divided by the 18 households. These data were presented in a heatmap created with the package ggplot2 in R, version 4.0.2 [36].

#### In-depth interview data collection methods

At the end of the second day of the direct observations, enumerators conducted a semi-structured interview with the primary child caregivers in each of these same 18 households. We developed the interview guide for the purposes of this study based on the guiding research question: “what beliefs and factors underlie the adoption of different chicken management practices?” The interview guide is available in Supplement 1.

#### In-depth interview data analysis

Transcripts from in-depth interviews were analyzed using thematic analysis, with a hybrid approach of both inductive and deductive coding [37] using the steps outlined by Braun and Clarke [38]. Text analysis was conducted using NVivo version 12. After reading the 18 transcripts, reviewing interview summaries from the research assistants, and taking memos concurrently to identify emerging concepts, initial codes were developed, and then the coded text was sorted into themes and subthemes. The codebook and coded transcripts were audited and validated by a second co-author (RA), and the full codebook has been included in Supplement 3. The 18 observations were also compared using data matrices, and the frequency of topics raised was observed using the text analysis features of NVivo. These themes and subthemes were linked back to the research questions and exemplar quotes were identified for each of these themes.

## Results

### Quantitative results

Table 1 presents demographic characteristics of the 973 households that raised at least one chicken. Notably, more than half of women in the sample had no formal education and most households had between five and seven household members. Nearly 80% of households had access to an improved water source but only 30% had improved sanitation services. On average, households had approximately 11 chickens and two other non-poultry livestock. Animal feces were visible on the property in about two-thirds of households, and 62% of index children observed had visibly clean hands.

**Table 1:** Demographic characteristics of households raising chickens in the Oromia and Amhara regions

		Total
		***N*** = 973
Region	Amhara	48.6% (473)
	Oromia	51.4% (500)
Agroecology	Lowland	37.1% (361)
	Midland	44.4% (432)
	Highland	18.5% (180)
Maternal age	15–30	40.4% (393)
	31–38	30.5% (297)
	39+	29.1% (283)
Household is woman-headed		11.1% (108)
Baseline education of woman	No schooling	55.8% (543)
	Primary 1	18.0% (175)
	Primary 2	12.5% (122)
	Secondary 1,2 or university	4.9% (48)
	Other	4.7% (46)
	Missing	4.0% (39)
Number of household members	1–4	19.9% (194)
	5–7	50.6% (492)
	8+	29.5% (287)
Number of chickens owned	1–3 chickens	41.4% (403)
	4–9 chickens	25.1% (244)
	10+ chickens	33.5% (326)
Number of other livestock		2.1 (0.8)
Has improved water		79.8% (776)
Has improved sanitation		29.9% (291)
No feces visible on property		32.6% (317)
Child’s hands are clean		62.4% (267)

Data are presented as mean (SD) for continuous measures, and % (n) for categorical measures. The variable for whether the child’s hands were clean was only calculated among the 428 households for with this was observed among the 431 households with an index child

Table 2 presents means of the exposure variables describing chicken management practices and both adjusted and unadjusted regressions examining specifically which practices were most strongly associated with measures of exposure to contamination in this population. Three households did not have observations for the outcome measure of whether the child’s hands were clean, resulting in a total sample of 428 children among households with a chicken coop and 348 among households without a chicken coop. While most households had some form of chicken coop (84%), the majority of coops were not enclosed, and there was substantial variation in the location and type of housing. Forty-five percent of coops were reported to be either inside or attached to the house (0 m from the household), and 50% of them were considered “open housing”, where chickens can come and go freely. Only 37% of households reported that no chickens slept inside the house in the previous night.

**Table 2:** Regression results describing the association between chicken management practices and measures of exposure to environmental contamination

		Outcome Variables			
		No feces visible		Child’s hands are clean	
Exposure Variables		Unadjusted	Adjusted	Unadjusted	Adjusted
	% (n)				
***Among all households***					
Has a chicken coop		0.65***	0.70***	0.93	0.94
	83.7 (814)	(0.52–0.82)	(0.55–0.91)	(0.78–1.10)	(0.80–1.09)
Number of chickens					
*Referent: Category = 1, 1–3 chickens*	41.4 (403)				
*Category = 2, 4–9 chickens*	25.1 (244)	0.96	1.01	0.92	0.93
		(0.78–1.18)	(0.83–1.23)	(0.76–1.12)	(0.75–1.15)
*Category = 3, 10+ chickens*	33.5 (326)	0.76**	0.79**	0.99	0.97
		(0.60–0.98)	(0.65–0.95)	(0.85–1.17)	(0.81–1.17)
Chickens did not sleep in house last night	37.6 (366)	1.82***	1.66***	1.12	1.03
		(1.39–2.37)	(1.22–2.25)	(0.96–1.31)	(0.89–1.19)
***Among households with a chicken coop***					
Type of coop					
*Referent: open housing*	49.8 (405)				
*Enclosed housing*	38.3 (312)	1.80***	1.83***	1.06	1.08***
		(1.41–2.30)	(1.45–2.32)	(0.84–1.34)	(1.07–1.10)
*Fencing*	6.4 (52)	1.30	1.54	1.21	1.42***
		(0.71–2.36)	(0.90–2.63)	(0.93–1.57)	(1.32–1.52)
*Other*	5.5 (45)	1.40	1.65	1.13	1.27**
		(0.68–2.89)	(0.90–3.03)	(0.86–1.49)	(1.05–1.54)
Distance from house to coop					
*Referent: 0 meters*	44.5 (362)				
*1–3 meters*	29.1 (237)	1.83***	1.56***	1.26**	1.29**
		(1.33–2.52)	(1.19–2.05)	(1.05–1.51)	(1.04–1.61)
*4+ meters*	26.4 (215)	1.43*	1.37*	1.23*	1.24
		(0.96–2.13)	(0.98–1.88)	(0.98–1.53)	(0.95–1.63)

*** *p* < 0.01, ** *p* < 0.05, * *p* < 0.1. Risk ratios shown, with robust 95% confidence intervals clustered at the village level in parentheses. Control variables in adjusted regressions included: region, agroecology, treatment group, wealth quintile, woman-headed household, tertiles of number of household members, tertiles of the number of livestock owned (excluding chickens), education category of woman of reproductive age, tertiles of women’s empowerment in chicken production score, education category of household head, improved water, improved sanitation. The outcome of whether the child’s hands are clean is among a subsample of households that had an index child 0–36 months of age

Having any kind of chicken coop was associated with a 30% increased risk of animal feces being observed on the compound in adjusted analyses. Having 10 or more chickens also reduced the risk of observing animal feces on the property, but neither the number of chickens nor having a chicken coop was associated with child cleanliness. Chickens sleeping inside of the house the previous night was found to be negatively associated with proxy measures of contamination.

Among households with a chicken coop, having enclosed animal housing (relative to open housing) improved the likelihood of no feces being observed by 1.83 times in adjusted models and increased the likelihood of children’s hands being clean by 1.08 times. In fact, all types of chicken coops other than open housing were associated with an increased likelihood of the index child having clean hands. Chicken housing located at least 1 m away from the household (relative to being attached to or inside of the house) was also significantly positively associated with the two outcome measures of cleanliness.

### Qualitative results

#### Direct observation results

**Fig. 2:**
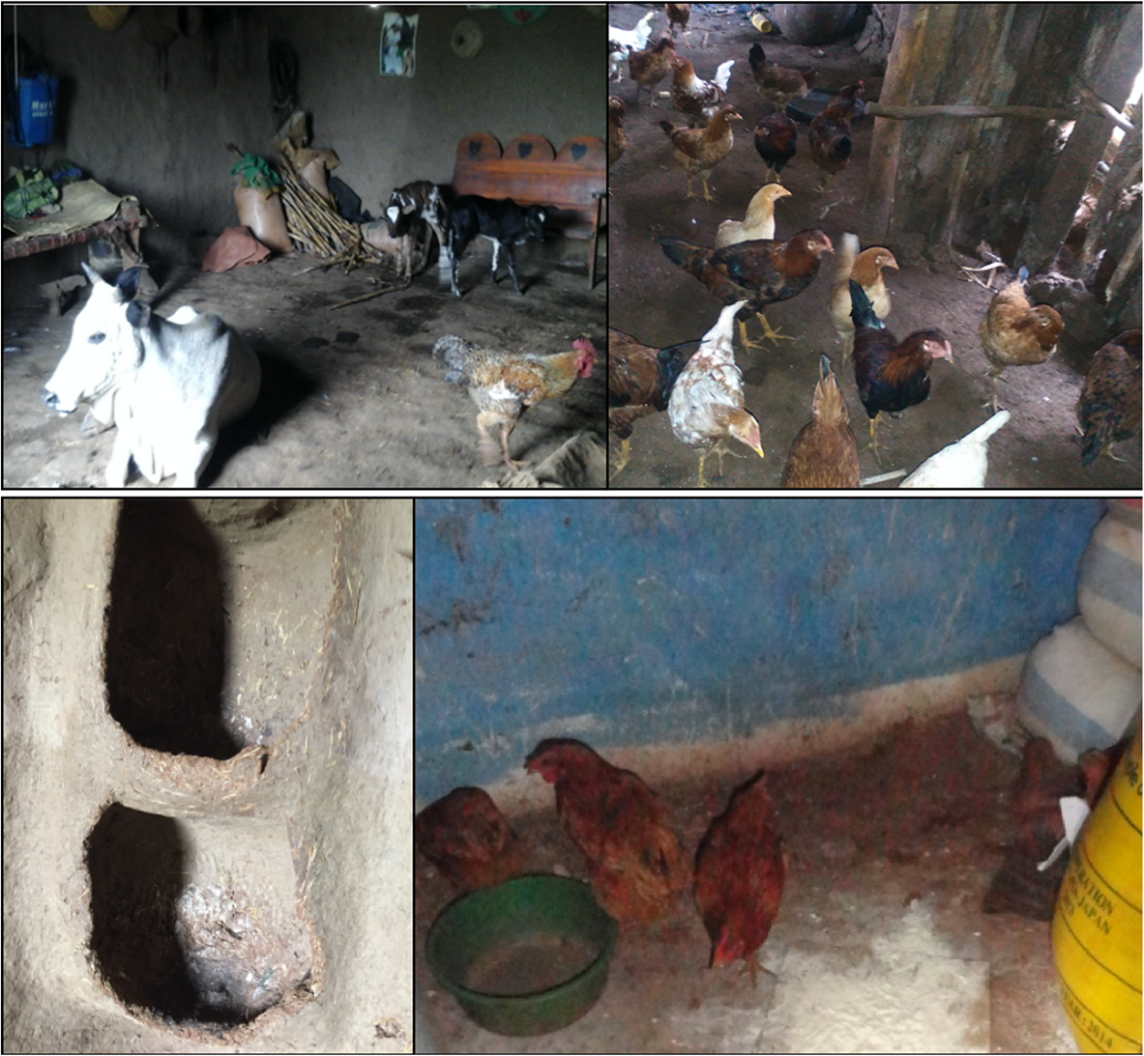
Locations of animal housing documented by enumerators during direct observations of 18 households with young children. Legend: Photos were taken by the following researchers and permission to use them has been obtained: top left: Mehfira Abdelmenan; bottom left: Ramya Ambikapathi; top right: Birki Gurmessa; bottom right: Amen Tesema. Top row: chickens sleep in the main house with other young livestock (left); separate chicken coop with open entry (right). Bottom row: chicken coop in a dugout earthen compartment of the interior house wall (left); chickens sleep in the grain store room (right)

Eighteen households were observed and interviewed. None of these households had a finished floor—all floors were made out of dung or dirt—and none had a handwashing station with soap. Only three households had a female index child, despite relative balance in the overall household sample. The mean age of the index children among these households was 23 months (SD 11). Sixty-one percent of households had their own latrine, and 72% had access to a standpipe or borehole for their drinking water, but only two households had an observed handwashing station (without soap). Only seven of the 18 households had a designated chicken coop at the time of observation. In 10 cases, the chickens usually spent the night inside the main house, and for three additional cases, they slept inside the house but in a separate room. As part of the direct observations, enumerators also took photos of where chickens spent the night, and observed substantial variation (Fig. 2). Chicken housing was observed inside or connected to the main house (along with other animals), in separate but open structures, inside of grain stores, connected to or inside of kitchens, and in only one case, in a confined and separated structure. These findings of substantial variation in the formality, types, and location of chicken housing mirror the results previously described in the quantitative data.

**Fig. 3:**
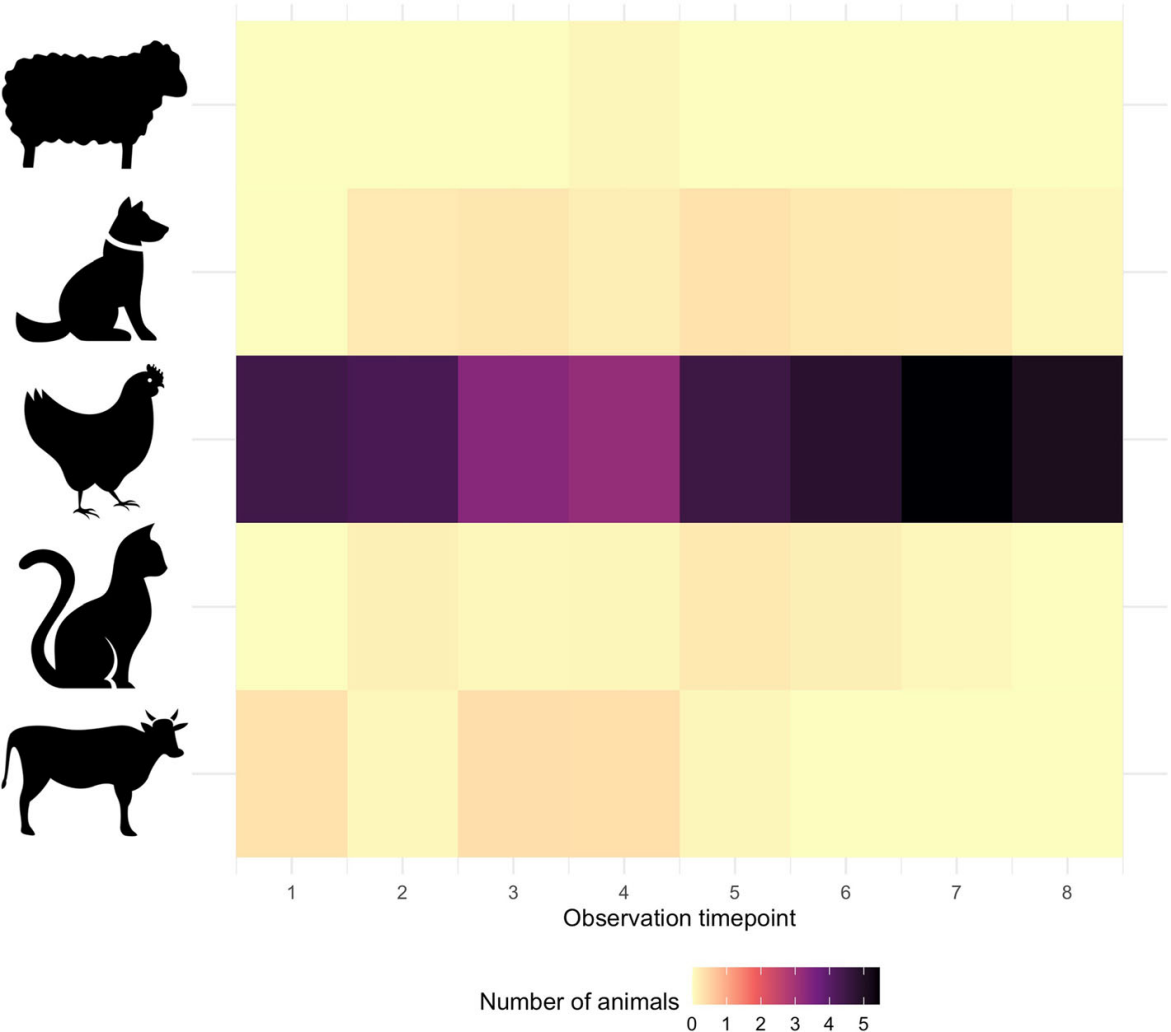
Number and type of animals observed inside of households during 6 h of observation. Legend: This figure shows the average number of different types of animals that were present inside of households during the 6 h of observation for the 18 houses observed. The observation period took place for 3 h per day over the course of 2 days. The observation timepoints 1–4 depicted on the X-axis correspond to tallies conducted after 0, 1, 2, and 3 h of observation on the first day; timepoints 4–8 correspond to tallies conducted after 0, 1, 2, and 3 h of observation on the second day. At each of these timepoints, enumerators tallied how many and what types of animals were observed inside of the household; the mean across all 18 households for each of the five animal types is depicted, corresponding to the darkness of the shading. The types of animals tallied are expressed on the y-axis, and include (from top to bottom include): sheep, dogs, chickens, cats, and bovines (cows/bulls/oxen)

During the eight instances of spot checks over the course of two days, enumerators observed that on average, animal feces were on the kitchen floor 30% of the time, there were an average of 2.8 chickens (SD 2.8) within five steps of the index child, there were feces visible within five paces of the index child 43% of the time, and animals were observed inside of the house 70% of the time. On average, there were 3.1 (SD 3.7) animals inside of a house at any given time across the 18 households. Figure 3 illustrates the density of different types of animals observed over the 12 h of observation, averaged across the 18 households observed. These results indicate two key findings. First, it is clear that other animals in addition to chickens were frequently kept inside of houses, including livestock (cows/oxen and sheep), but also domestic animals such as cats and dogs. Second, chickens were by far the most commonly observed animal inside the house —at the average rate of 3 to 5 chickens at any given time—and they wandered in and out throughout the period of direct observation.

Table 3 presents the results of behaviors observed during the 6 h of observation for the 18 households. Enumerators observed potentially harmful behaviors—including the child putting fingers in their mouth, dirt in their mouth, a dirty object in their mouth, touching an animal, or eating leftover uncovered food—in 100% of households, at an average rate of 7 times per hour. Protective behaviors—including handwashing of the index child’s or caregiver’s hands with and/or without soap—were observed much less commonly, among 78% of households at a rate of only 0.5 times per hour. Overall, handwashing with soap was uncommon, and observed in less than a quarter of households for caregivers and only one-sixth of households for children.

**Table 3:** Child exposures observed over 6 h of household observation with 18 households with young children

	% of households in which behavior was observed	Average # of times per hour
Harmful behaviors	100.0	7.0
*Children’s fingers in mouth*	100.0	3.0
*Dirt in mouth*	83.3	0.9
*Dirty object in mouth*	94.4	2.5
*Touched animal*	66.7	0.5
*Ate leftover uncovered food*	38.9	0.1
Protective behaviors	77.8	0.5
*Child’s hands were washed with soap*	16.7	0.0
*Child’s hands were washed without soap*	66.7	0.3
*Woman caregiver washed hands with soap*	22.2	0.1
*Woman caregiver washed hands without soap*	61.1	2.3

#### Results from in-depth interviews with primary woman caregiver

In the text analysis, 55 codes were developed and used to identify three major themes: 1) protection of animals from threats, 2) maintaining cleanliness and health of the household, and 3) constraints to the adoption of ideal management practices. These themes are described in detail below, using exemplar quotes to highlight key points. We also indicate the treatment arm associated with each quote to highlight any differences that might have occurred due to interventions, since the qualitative study was conducted during the midline evaluation of the trial.

##### Protection of animals

Whether households kept their chickens in a separated coop or inside of their home, most respondents named protection from various threats as the primary reason underlying the location. There were several threats that were commonly noted across households, especially predators (*n* = 14) and theft (*n* = 3). Several households also mentioned risk of the chickens wandering off, destroying crops or the neighbor’s land, being trampled by larger livestock, or hurting and being hurt by children:

“We fear to lose [them] if chickens go outside, because there are so many things that can hunt and harm our chickens out there. We let them roam around and take air for a short time...then we gather and return them back into their corral” (Respondent 22, ACGG).

Respondents often recognized the benefits of allowing chickens to roam freely to forage for food or get fresh air, which were both seen as beneficial for the health and egg productivity of the birds. However, this view was often in conflict with the dangers of predators:

“It would be better for the chickens to roam around because this way they are able to find and pick their own food. But if we leave them freely, they will be eaten by wild beasts” (Respondent 19, ACGG).

Lastly, protection of the hybrid chickens specifically was an important determinant of practices among intervention households, since these birds were seen as being both more valuable and more vulnerable compared to local varieties. As one respondent described, “it is okay for the local ones to roam freely in the winter but not the hybrid ones … to protect them from beasts” (Respondent 16, ACGG). This tendency to treat the hybrid chickens differently than local chickens was also noted in where the chickens were kept. There were several cases where hybrid chickens were kept inside the main house while local chickens were allowed to roam more freely, since hybrid chickens were seen as slower, less resilient, more valuable, and more prone to loss.

##### Maintaining cleanliness and/or health

Most respondents associated cleanliness with household health, but there were often misconceptions about the pathways through which chickens could adversely affect health. For example, households often listed the smell (*n* = 10), dirtiness of the chickens (*n* = 13), feces (*n* = 17), and/or the family’s health in relation to the environment (*n* = 16) as reasons to separate chickens from the household or as consequences of chicken production. Smell was often viewed as a source of illness; as one respondent described, “cattle and humans don’t have to live together, because their feces’ smell cause disease” (Respondent 41, ATONU). Two households mentioned maintaining cleanliness aesthetically, for a more comfortable lifestyle:

“It is not matched with our living style. Having them at home with the family is an odious thing. To make the situation comfortable, it is better to have them far from the cooking facility or kitchen and clean the place frequently as to not affect them as well as to make the place interesting” (Respondent 56, Control).

While about two-thirds of households mentioned trying to keep children and chickens away from each other, only about half of households stated a reason related to sanitation and hygiene, while the other half were more likely to list physical reasons for keeping them separated. For example:

“Chickens may not affect child, rather child may affect chickens. But larger chickens may bite the [eyes of the] child, so that I may protect one from each other but chicken and child can freely play together” (Respondent 55, Control);

Seven households mentioned reasons for separation that were either related to dirtiness or contagious insects, and five specifically mentioned issues related to illness from contact with chicken waste:

“The child can catch disease if it gets into the chicken coop and touches the waste of chickens with [their] hand. It may even go on to put the dirt from the chicken coop into its mouth— this is dangerous for the health of the child” (Respondent 22, ACGG).

##### Constraints to adopting ideal practices

Although 13 respondents recognized either cleanliness or health issues related to where chickens were kept, they were not always able to adopt more permanent solutions due to a number of constraints. One constraint commonly listed was feed. While 12 households mentioned the benefits of cooping chickens, 10 mentioned that access to feed, especially for hybrid varieties of chickens that require more food, is a challenge. Allowing chickens to roam at several points in the day grants the opportunity for them to forage for their own food, which reduces both the financial and time cost of feeding them:

“They have great advantage, but many of them died, plus the hybrid chickens need too much food. They only give us eggs when they eat well” (Respondent 16, ACGG).

The type of management practices adopted was also tied to the number of chickens being raised and the age of the chickens. If a household had fewer chickens, they were more likely to keep them inside and abandon the use of a chicken coop because it did not seem worthwhile to build a separate structure when they do not take up much space:

“Because [the chickens] are few, they stay with us here … I can’t think of a better place other than this!” (Respondent 17, Control).

The chicks were seen as more vulnerable to harm, and thus might be kept inside while they grow. As one responded recalled, “they told us they should get warm so I let them stay in the house till they grow; now they have feathers” (Respondent 16, ACGG). Lastly, not having enough space (*n* = 2), time (*n* = 1), resources (n = 1), or household bargaining power with a husband (n = 2) to build a coop were seen as barriers to adopting coops for some households.

## Discussion

Both our quantitative and qualitative findings support that children’s physical exposure to chickens is high among households raising chickens in these two regions of Ethiopia, and that specific chicken husbandry practices were linked to increased exposure to environmental contamination. The frequency and proximity of exposure pose substantial health risks for this population, and especially for households with young children, who are most vulnerable to adverse health and nutrition consequences.

To synthesize our findings, first, our quantitative results demonstrated that as the number of chickens raised increased, exposure to animal feces actually decreased. While having any chicken coop increased the risk of observing animal feces—likely because this was a proxy for raising enough chickens to warrant a coop—it was clear that not all coops were equally protective. Thus, while more animals can potentially equate to increased levels of contamination, it also appears that this may be somewhat compensated by increased measures of protection. This trend was supported by both phases of our qualitative research. Household observations and interviews revealed that if there were fewer chickens, or after high rates of chicken mortality, households often abandoned the use or upkeep of a coop and instead kept chickens inside the home. From this interpretation, having fewer chickens could potentially be associated with higher exposure to contamination, if it is accompanied by more informal animal husbandry. However, it is worth noting that this study selected for small-scale chicken producers (the median number of chickens owned was 6), so we have fewer observations to determine the full spectrum of this relationship, and whether it is truly negative or possibly U-shaped.

Second, we found that the type and location of poultry housing used were significantly associated with exposure to environmental contamination in the household environment. Our results showed a strong relationship between the distance of chicken housing from the house and risk of children’s hands being dirty and observing animal feces on the property. While we only used proxy measures of exposure to contamination, previous literature has shown that keeping chickens in the house was negatively associated with environmental enteropathy [39] and lower height-for-age z-scores in children [22]. Among households with coops, the strongest predictors of reducing exposure to contamination were having a coop that is separated from the household and having enclosed chicken housing. It is notable that many of these practices reduced the risk of observing animal feces on the property, since the presence of visible animal feces has been associated with an increased risk of diarrheal disease for young children [7]. These findings suggest having coops that are enclosed and located a safe distance away from the household could potentially reduce the health risks of household members.

Third, a major contribution of this study was to identify the nuance that was not captured by proxy measures of exposure—for example, the variation in what households considered to be a “coop.” These findings can help to inform future data collection. Since simply asking about whether or not a coop exists might not be an adequate measure of animal husbandry or level of exposure to environmental contamination, researchers should consider asking about scavenging practices (is there an enclosed grazing corral, or do chickens scavenge freely?), where chickens sleep at night, location of a chicken coop relative to the main house (is it inside of a kitchen or grain store? Inside the main house or sleeping room?), type of structure, and whether or not the coop is enclosed. Our results clearly show that while having a coop might act as a proxy for an adverse health exposure, more detail regarding the type of coop can indicate protective practices.

Fourth, from the direct observation results, we showed that young children were directly affected by animal mobility and environmental conditions. Animals were frequently found inside the house and/or next to children. In two-thirds of households, index children directly touched an animal, and in 83% of households children put dirt in their mouths. As previous research has shown, in settings where animals are allowed to roam freely it is highly likely that the dirt to which children are exposed contains harmful pathogens [14, 40]. Although we observed some potential behavioral compensation in the form of handwashing, most handwashing occurred without soap and thus was unlikely to fully eliminate any harmful pathogens. Overall, these behaviors demonstrated that children living in chicken-producing households faced regular exposure to contamination in this context, especially when animals and children were not physically separated.

Lastly, our results highlighted the factors that drive the adoption (or the lack of adoption) of specific chicken husbandry practices. While households participating in the ACGG intervention were more likely to have a chicken coop due to the intervention encouraging this practice, the qualitative data revealed a number of factors that influenced where the coop was located and how it was used in practice. Perceived threats of predation, other animals, theft, loss, and destruction of crops can drive households to keep their poultry either in a coop or inside of the household. While the majority of households recognized the potential health, cleanliness, or physical harm of animals staying in the house or in close proximity to children, there were a number of factors that prevented women from adopting different practices, especially access to feed and resource constraints. Analysis of the interview data by treatment group highlighted how the ATONU group appeared to be more sensitized to the threats of exposure to contamination, although households were not necessarily equipped with the resources to act on these perceptions. While we recognize that decision making is only one dimension of women’s empowerment, previous research from Indonesia, Bolivia, Peru, and Kenya has shown that women’s control over livestock and the productive resources needed to raise them has been associated with increased bargaining power, access to animal source foods that can benefit their children, and their empowerment [41]. Thus, poultry interventions should be designed specially with women’s empowerment as a goal in order to ensure their control over the benefits gained from production.

Previous research has also found high levels of fecal contamination from poultry in rural Ethiopia, and that contamination has been negatively associated with health and nutrition outcomes. Using spot checks and household survey data, the study by Headey et al. (2017) found that while poultry ownership was positively associated with child height-for-age z-scores [β = 0.291], the practice of corralling poultry inside the house overnight was negatively associated with height-for-age z-scores [β = − 0.250]. The authors also found no negative associations between HAZ and corralling other livestock species indoors [22]. A follow-up paper also found that the presence of animal feces on a household’s property was negatively associated with diarrhea and fever in Vietnam and Bangladesh, cough/cold in Vietnam, and child height-for-age z-scores in Bangladesh and Ethiopia [11]. As part of formative research for a household nutrition and WASH trial (the Sanitation, Hygiene, Infant Nutrition Efficacy Project, or SHINE trial), Ngure et al. (2013) observed the WASH behaviors and exploratory ingestion of infants in 23-caregiver-child pairs. All chicken feces sampled later tested positive for *E. coli*. *E. coli* were found on 30% of the dominant hands of caregivers and on infants’ left and right hands in 11 and 5% of cases, respectively. The paper estimated that a one-year-old child in rural Zimbabwe may typically consume up to one gram of chicken feces, 20 g of soil, and 400 mL of contaminated water per day. As a result, infants would ingest anywhere from 4.7 million to 23.0 million *E. coli* bacteria [14]. Together, these previous findings support the fact that our proxy measurements related to exposure to contamination are predictive of actual exposure levels, and that these levels are associated with adverse child nutrition and health outcomes.

Nonetheless, the ACGG+ATONU evaluation did not observe acute health risks associated with an exogenous increase in chicken production in this same population [26]. Using household survey data from 9 and 18 months of follow-up, Passarelli et al. explored the effectiveness of the African Chicken Genetic Gains (ACGG) chicken production intervention both with and without the additional ATONU nutrition promotion component. The authors observed a benefit of the ACGG intervention for children’s height-for-age and weight-for-age z-scores, but found no evidence of an increase in child morbidity [26]. Conversely, other research has shown that exposure to animal feces does lead to acute disease outcomes, and notably, that it can also lead to chronic conditions like environmental enteric dysfunction that were not assessed in the ACGG/ATONU trial.

Recent research from the Democratic Republic of the Congo (DRC) provides a useful mixed-methods perspective that builds upon the framework of Passarelli et al. to inform our findings. Kuhl et al. conducted formative research to understand the primary sources of exposure to fecal contamination in DRC and to develop theory-driven and evidence-based interventions to reduce these exposures. To design their research activities, the authors applied the IBM-WASH framework [42] to consider how contextual, psychosocial, and technological factors influence health behaviors across multiple levels (structural, community, interpersonal, individual, and habitual) [24]. They found that while caregivers were often aware that children’s exposure to feces was an issue, a lack of caregiver time, financial resources, and enabling technologies (including safe child play spaces and enclosures for small animals) served as barriers [24]. Our qualitative findings similarly support the fact that, while the specific barriers to safe environmental conditions may vary across contexts, access to improved husbandry technologies can help to alleviate the resulting health risks.

Our results have several implications for policy and practice. First, there can be negative consequences related to projects that promote increases in animal production, especially if appropriate animal husbandry practices are not implemented. This is especially true in the context of semi-scavenging systems, where an increase in animals may result a higher concentration of roaming animals depositing feces. One solution might be to move away from semi-scavenging systems towards systems with enclosures. However, as the qualitative interviews suggested, one of the disadvantages of corralling animals is the increased requirement for food, water, and veterinary care, which are often not available or accessible in remote settings. Thus, any efforts to move away from semi-scavenging systems should ensure that these inputs are available and affordable.

Previous trials have tested the effectiveness of enclosed child play areas to reduce young children’s exposure to threats such as feces from animals, but these were not shown to be effective in reducing stunting, diarrhea [43], or enteric infections [44]. Based on our results, perhaps addressing animal husbandry practices such as the type and location of livestock housing could provide a health benefit. Several projects designed to test different methods of limiting exposure to contamination from poultry are currently already underway [20, 45]. In addition, training should be provided on the safe management and disposal of manure, given some research suggesting that chicken coops actually increase health risks, due to the potential for exposure to a higher concentration of manure [46].

Overall, our findings suggest the need for greater collaboration across the nutrition, health, livestock, and agricultural sectors. Integration of social and behavioral change communication for nutrition and health would benefit from including messaging on animals in contexts where livestock rearing is common, and that livestock interventions would benefit from a greater focus on practices that minimize exposure to humans. Moreover, animals should no longer be left out of traditional water, sanitation, and hygiene interventions. As Prendergast et al. (2019) argue, practitioners must put the “A”—for animals—into “WASH” for integrated management of water, animals, sanitation, and hygiene in public health interventions [47]. Movements such as “One Health” recognize the interconnectedness of human, animal, and environmental health, and the benefits that can be gained for all by working collaboratively across multiple disciplines [48].

This study has several limitations. These results are not generalizable to all rural populations, but specific to the study population of chicken-rearing farmers included in our analysis. In order to maximize the number of observations in our quantitative analyses, we did not restrict all analyses to households with young children. As a result, the relationships observed in the quantitative analyses may not be directly applicable to the households for which we have qualitative data, and vice versa for the qualitative findings. Moreover, we recognize that our regression results are based on cross-sectional data, and thus could be subject to confounding; we attempted to address this by controlling for a number of potential confounders, but we do not assert that the relationships we observe are causal, merely associative. In addition, this study does not measure contamination directly (for example, as *E. coli* counts), but rather uses proxy measures of contamination. While direct measurement was beyond the scope of this study, we recognize the limitation of these proxies for estimating specific types and levels of pathogens. Lastly, this study only explores how chicken management practices influence waste and does not account for other animal-related health risks, like other livestock, human excrement, physical harm, fungi, and insects.

Our analysis also has several strengths; namely, the mixed-methods approach supports the research questions from several different perspectives and data sources. Our qualitative sampling by treatment group also allowed us to observe differences in behaviors that might be influenced by the intervention, while also identifying commonalities in constraints across all households. In addition, the qualitative interview data helped shed light on practical considerations for implementers of nutrition-sensitive agriculture interventions by showing the constraints that households face when considering the adoption of alternative methods.

Based on our findings, practitioners promoting animal agriculture must thoroughly provide all of the knowledge and inputs required for a safe and sustainable animal production system. Household environments with limited WASH conditions might not be able to adequately absorb an intensification of animal agriculture. However, approximately 60% of rural households in low and middle-income countries depend on livestock for their livelihoods. Thus, the pertinent question is not whether livestock should continue to be raised on a small scale, but how to do so safely.

## Supplementary Information

**Additional file 1: Supplement 1.** Semi-Structured Interview Guide: In-Depth Interviews with Women. Supplemental file containing the full semi-structured interview guide for the in-depth interviews with women during the midline evaluation. This interview guide was developed by the authors of this manuscript for the purposes of this study.

**Additional file 1: Supplement 2.** Direct Household Observation Questionnaire. This file contains the full data collection instrument used for the direct observations of the 18 households included in the midline observation phase.

**Additional file 3: Supplement 3.** Codebook for Women’s In-depth Interviews. This supplement contains the codebook with all codes used to analyze the transcripts of the in-depth interviews using thematic analysis. These codes were exported from NVivo after analysis.

## Data Availability

These data are not publicly available at this time due to data sharing restrictions with project partners that prohibit their sharing.

## Abbreviations

WASH: Water, sanitation, and hygiene
SD: Standard deviation
CI: Confidence interval
EED: Environmental enteric dysfunction
HAZ: Height-for-age z-score
WAZ: Weight-for-age z-score
WHZ: Weight-for-height z-score

## References

[1] UNICEF/WHO/World Bank (2019). Malnutrition. UNICEF/WHO/World Bank Joint Child Malnutrition Estimates, March 2019 edition.

[2] Arimond M, Ruel MT (2004). Dietary diversity is associated with child nutritional status: evidence from 11 demographic and health surveys. J Nutr.

[3] Ruel MT, Quisumbing AR, Balagamwala M. Nutrition-sensitive agriculture: what have we learned so far? Global Food Secur. 2018;17:128-53.

[4] Leroy JL, Frongillo EA (2007). Can interventions to promote animal production ameliorate undernutrition?. J Nutr.

[5] Randolph TF, Schelling E, Grace D, Nicholson CF, Leroy JL, Cole DC (2007). Invited review: role of livestock in human nutrition and health for poverty reduction in developing countries.

[6] Masset E, Haddad L, Cornelius A, Isaza-Castro J (2012). Effectiveness of agricultural interventions that aim to improve nutritional status of children: systematic review. BMJ.

[7] Penakalapati G, Swarthout J, Delahoy MJ, McAliley L, Wodnik B, Levy K, Freeman MC (2017). Exposure to animal feces and human health: a systematic review and proposed research priorities. Environ Sci Technol.

[8] Matilla F, Velleman Y, Harrison W, Nevel M (2018). Animal influence on water, sanitation and hygiene measures for zoonosis control at the household level: a systematic literature review. PLoS Negl Trop Dis.

[9] Zambrano LD, Levy K, Menezes NP, Freeman MC (2014). Human diarrhea infections associated with domestic animal husbandry: a systematic review and meta-analysis. Trans R Soc Trop Med Hyg.

[10] Humphrey JH (2009). Child undernutrition, tropical enteropathy, toilets, and handwashing. Lancet.

[11] Headey D, Nguyen P, Kim S, Rawat R, Ruel M, Menon P. Is exposure to animal feces harmful to child nutrition and health outcomes? A multicountry observational analysis. Am J Tropical Med Hygiene. 2017;96(4):961-9. 10.4269/ajtmh.16-0270 PMC5392649 27994099

[12] Ngure FM, Reid BM, Humphrey JH, Mbuya MN, Pelto G, Stoltzfus RJ (2014). Water, sanitation, and hygiene (WASH), environmental enteropathy, nutrition, and early child development: making the links. Ann N Y Acad Sci.

[13] George CM, Cirhuza LB, Kuhl J, Williams C, Coglianese N, Thomas E (2021). Child Mouthing of Feces and Fomites and Animal Contact are Associated with Diarrhea and Impaired Growth Among Young Children in the Democratic Republic of the Congo: A Prospective Cohort Study (REDUCE Program). J Pediatr.

[14] Ngure FM, Humphrey JH, Mbuya MNN, Majo F, Mutasa K, Govha M (2013). Formative research on hygiene behaviors and geophagy among infants and young children and implications of exposure to fecal bacteria. Am J Trop Med Hyg.

[15] Ercumen A, Pickering AJ, Kwong LH, Arnold BF, Parvez SM, Alam M, et al. Animal feces contribute to domestic fecal contamination: evidence from *E. coli* measured in water, hands, food, flies, and soil in Bangladesh. Environ Sci Technol. 2017;51(15):8725-34. 10.1021/acs.est.7b01710 PMC5541329 28686435

[16] Marquis GS, Ventura G, Gilman RH, Porras E, Miranda E, Carbajal L, Pentafiel M (1990). Fecal contamination of shanty town toddlers in households with non-corralled poultry, Lima. Peru American Journal of Public Health.

[17] Pickering A, Julian T. Fecal contamination and diarrheal pathogens on surfaces and in soils among Tanzanian households with and without improved sanitation. Sci technol. 2012;46(11):5736-43. 10.1021/es300022c 22545817

[18] Keusch GT, Denno DM, Black RE, Duggan C, Guerrant RL, Lavery JV (2014). Environmental Enteric Eysfunction : Pathogenesis , Diagnosis and Clinical Consequences. Clin Infect Dis.

[19] Humphrey J, Jones A. The Sanitation Hygiene Infant Nutrition Efficacy (SHINE) Trial: Rationale, Design, and Methods. Clin Infect. 2015;61(Suppl 7):S685-702. 10.1093/cid/civ844 PMC4657589 26602296

[20] Gelli A, Headey D, Becquey E, Ganaba R, Huybregts L, Pedehombga A, Santacroce M, Verhoef H (2019). Poultry husbandry, water, sanitation, and hygiene practices, and child anthropometry in rural Burkina Faso. Matern Child Nutri.

[21] Arnold BF, Null C, Luby SP, Unicomb L, Stewart CP, Dewey KG, Ahmed T, Ashraf S, Christensen G, Clasen T, Dentz HN, Fernald LCH, Haque R, Hubbard AE, Kariger P, Leontsini E, Lin A, Njenga SM, Pickering AJ, Ram PK, Tofail F, Winch PJ, Colford JM (2013). Cluster-randomised controlled trials of individual and combined water, sanitation, hygiene and nutritional interventions in rural Bangladesh and Kenya: the WASH benefits study design and rationale. BMJ Open.

[22] Headey D, Hirvonen K. Is exposure to poultry harmful to child nutrition? An observational analysis for rural Ethiopia. PLoS ONE. 2016;11(8):e0160590. 10.1371/journal.pone.0160590 PMC4986937 27529178

[23] Harvey SA, Winch PJ, Leontsini E, Torres Gayoso C, López Romero S, Gilman RH, Oberhelman RA (2003). Domestic poultry-raising practices in a Peruvian shantytown: implications for control of campylobacter jejuni-associated diarrhea. Acta Trop.

[24] Kuhl J, Bisimwa L, Thomas ED, Williams C, Ntakirutimana J, Coglianese N (2021). Formative research for the development of baby water, sanitation, and hygiene interventions for young children in the Democratic Republic of the Congo (REDUCE program). BMC Public Health.

[25] Ngure F, Gelli A, Becquey E, Ganaba R, Headey D, Huybregts L, Pedehombga A, Sanou A, Traore A, Zongo F, Zongrone A (2019). Exposure to livestock feces and water quality, sanitation, and hygiene (wash) conditions among caregivers and young children: formative research in rural Burkina Faso. Am J Trop Med Hyg.

[26] Passarelli S, Ambikapathi R, Gunaratna NS, Madzorera I, Canavan CR, Noor AR, Worku A, Berhane Y, Abdelmenan S, Sibanda S, Munthali B, Madzivhandila T, Sibanda LM, Geremew K, Dessie T, Abegaz S, Assefa G, Sudfeld C, McConnell M, Davison K, Fawzi W (2020). A chicken production intervention and additional nutrition behavior change component increased child growth in Ethiopia: a cluster-randomized trial. J Nutr.

[27] Herforth A, Harris J (2014). Understanding and Applying Primary Pathways and Principles. Brief #1.

[28] Creswell JW (2015). A concise introduction to mixed methods research.

[29] Fetters MD, Curry LA, Creswell JW (2013). Achieving integration in mixed methods designs - Principles and practices. Health Services Research.

[30] Nguyen PH, Headey D, Frongillo EA, Tran LM, Rawat R, Ruel MT, et al. Changes in underlying determinants explain rapid increases in child linear growth in Alive & Thrive Study Areas between 2010 and 2014 in Bangladesh and Vietnam. J Nutr. 2017:jn243949. 10.3945/jn.116.243949. 10.3945/jn.116.243949 PMC5320405 28122930

[31] Agriculture to Nutrition (ANTONU): Evaluation of Integrated Agriculture and Nutrition-Sensitive Interventions for the African Chicken Genetic Gains (ACGG) Program in Ethiopia - Baseline Data. Africa Portal. 2017. https://www.africaportal.org/publications/agriculture-nutrition-antonu-evaluation-integrated-agriculture-and-nutrition-sensitive-interventions-african-chicken-genetic-gains-acgg-program-ethiopia-baseline-data/. Accessed 6 May 2021.

[32] Dessie T, Ogle B. Village poultry production systems in the central highlands of Ethiopia. Trop Anim Health Prod. 2001;33(6):521-37. 10.1023/a:1012740832558 11770206

[33] Halima H, Neser FWC, Van Marle-Koster E. De Kock A. Tropical Animal Health and Production: Village-based indigenous chicken production system in north-west Ethiopia. 2007;39(3):189-97. 10.1007/s11250-007-9004-6 17691543

[34] WHO, UNICEF, USAID (2015). WHO | improving nutrition outcomes with better water, sanitation and hygiene: practical solutions for policy and programmes.

[35] Mooney CZ, Mooney CF, Mooney CL, Duval RD, Duvall R. Bootstrapping: a nonparametric approach to statistical inference. SAGE; 1993.

[36] R Development Core Team (2017). R: A language and environment for statistical computing. Vienna, Austria.

[37] Fereday J, Muir-Cochrane E (2006). Demonstrating rigor using thematic analysis: a hybrid approach of inductive and deductive coding and theme development. Int J Qual Methods.

[38] Braun V, Clarke V (2006). Using thematic analysis in psychology. Qual Res Psychol.

[39] George CM, Oldja L, Biswas SK, Perin J, Lee GO, Ahmed S, et al. Fecal markers of environmental enteropathy are associated with animal exposure and caregiver hygiene in Bangladesh. Am J Trop Med Hygiene. 2015;93(2):269-75. 10.4269/ajtmh.14-0694 PMC4530746 26055734

[40] Pickering AJ, Ercumen A, Arnold BF, Kwong LH, Parvez SM, Alam M, Sen D, Islam S, Kullmann C, Chase C, Ahmed R, Unicomb L, Colford JM, Luby SP (2018). Fecal Indicator Bacteria along multiple environmental transmission pathways (water, hands, food, soil, flies) and subsequent child diarrhea in rural Bangladesh. Environ Sci Technol.

[41] Valdivia C. Gender, livestock assets, resource management, and food security: lessons from the SR-CRSP. Agric Hum Values. 2001;18(1):27-39.

[42] The Integrated Behavioural Model for Water, Sanitation, and Hygiene: a systematic review of behavioural models and a framework for designing and evaluating behaviour change interventions in infrastructure-restricted settings - PubMed. https://pubmed.ncbi.nlm.nih.gov/24160869/. Accessed 23 Apr 2021. 10.1186/1471-2458-13-1015 PMC4231350 24160869

[43] Humphrey JH, Mbuya MNN, Ntozini R, Moulton LH, Stoltzfus RJ, Tavengwa NV, Mutasa K, Majo F, Mutasa B, Mangwadu G, Chasokela CM, Chigumira A, Chasekwa B, Smith LE, Tielsch JM, Jones AD, Manges AR, Maluccio JA, Prendergast AJ, Humphrey JH, Jones AD, Manges A, Mangwadu G, Maluccio JA, Mbuya MNN, Moulton LH, Ntozini R, Prendergast AJ, Stoltzfus RJ, Tielsch JM, Chasokela C, Chigumira A, Heylar W, Hwena P, Kembo G, Majo FD, Mutasa B, Mutasa K, Rambanepasi P, Sauramba V, Tavengwa NV, van der Keilen F, Zambezi C, Chidhanguro D, Chigodora D, Chipanga JF, Gerema G, Magara T, Mandava M, Mavhudzi T, Mazhanga C, Muzaradope G, Mwapaura MT, Phiri S, Tengende A, Banda C, Chasekwa B, Chidamba L, Chidawanyika T, Chikwindi E, Chingaona LK, Chiorera CK, Dandadzi A, Govha M, Gumbo H, Gwanzura KT, Kasaru S, Makasi R, Matsika AM, Maunze D, Mazarura E, Mpofu E, Mushonga J, Mushore TE, Muzira T, Nembaware N, Nkiwane S, Nyamwino P, Rukobo SD, Runodamoto T, Seremwe S, Simango P, Tome J, Tsenesa B, Amadu U, Bangira B, Chiveza D, Hove P, Jombe HA, Kujenga D, Madhuyu L, Makoni PM, Maramba N, Maregere B, Marumani E, Masakadze E, Mazula P, Munyanyi C, Musanhu G, Mushanawani RC, Mutsando S, Nazare F, Nyarambi M, Nzuda W, Sigauke T, Solomon M, Tavengwa T, Biri F, Chafanza M, Chaitezvi C, Chauke T, Chidzomba C, Dadirai T, Fundira C, Gambiza AC, Godzongere T, Kuona M, Mafuratidze T, Mapurisa I, Mashedze T, Moyo N, Musariri C, Mushambadope M, Mutsonziwa TR, Muzondo A, Mwareka R, Nyamupfukudza J, Saidi B, Sakuhwehwe T, Sikalima G, Tembe J, Chekera TE, Chihombe O, Chikombingo M, Chirinda T, Chivizhe A, Hove R, Kufa R, Machikopa TF, Mandaza W, Mandongwe L, Manhiyo F, Manyaga E, Mapuranga P, Matimba FS, Matonhodze P, Mhuri S, Mike J, Ncube B, Nderecha WTS, Noah M, Nyamadzawo C, Penda J, Saidi A, Shonhayi S, Simon C, Tichagwa M, Chamakono R, Chauke A, Gatsi AF, Hwena B, Jawi H, Kaisa B, Kamutanho S, Kaswa T, Kayeruza P, Lunga J, Magogo N, Manyeruke D, Mazani P, Mhuriyengwe F, Mlambo F, Moyo S, Mpofu T, Mugava M, Mukungwa Y, Muroyiwa F, Mushonga E, Nyekete S, Rinashe T, Sibanda K, Chemhuru M, Chikunya J, Chikwavaire VF, Chikwiriro C, Chimusoro A, Chinyama J, Gwinji G, Hoko-Sibanda N, Kandawasvika R, Madzimure T, Maponga B, Mapuranga A, Marembo J, Matsunge L, Maunga S, Muchekeza M, Muti M, Nyamana M, Azhuda E, Bhoroma U, Biriyadi A, Chafota E, Chakwizira A, Chamhamiwa A, Champion T, Chazuza S, Chikwira B, Chingozho C, Chitabwa A, Dhurumba A, Furidzirai A, Gandanga A, Gukuta C, Macheche B, Marihwi B, Masike B, Mutangandura E, Mutodza B, Mutsindikwa A, Mwale A, Ndhlovu R, Nduna N, Nyamandi C, Ruvata E, Sithole B, Urayai R, Vengesa B, Zorounye M, Bamule M, Bande M, Chahuruva K, Chidumba L, Chigove Z, Chiguri K, Chikuni S, Chikwanda R, Chimbi T, Chingozho M, Chinhamo O, Chinokuramba R, Chinyoka C, Chipenzi X, Chipute R, Chiribhani G, Chitsinga M, Chiwanga C, Chiza A, Chombe F, Denhere M, Dhamba E, Dhamba M, Dube J, Dzimbanhete F, Dzingai G, Fusira S, Gonese M, Gota J, Gumure K, Gwaidza P, Gwangwava M, Gwara W, Gwauya M, Gwiba M, Hamauswa J, Hlasera S, Hlukani E, Hotera J, Jakwa L, Jangara G, Janyure M, Jari C, Juru D, Kapuma T, Konzai P, Mabhodha M, Maburutse S, Macheka C, Machigaya T, Machingauta F, Machokoto E, Madhumba E, Madziise L, Madziva C, Madzivire M, Mafukise M, Maganga M, Maganga S, Mageja E, Mahanya M, Mahaso E, Mahleka S, Makanhiwa P, Makarudze M, Makeche C, Makopa N, Makumbe R, Mandire M, Mandiyanike E, Mangena E, Mangiro F, Mangwadu A, Mangwengwe T, Manhidza J, Manhovo F, Manono I, Mapako S, Mapfumo E, Mapfumo T, Mapuka J, Masama D, Masenge G, Mashasha M, Mashivire V, Matunhu M, Mavhoro P, Mawuka G, Mazango I, Mazhata N, Mazuva D, Mazuva M, Mbinda F, Mborera J, Mfiri U, Mhandu F, Mhike C, Mhike T, Mhuka A, Midzi J, Moyo S, Mpundu M, Msekiwa N, Msindo D, Mtisi C, Muchemwa G, Mujere N, Mukaro E, Muketiwa K, Mungoi S, Munzava E, Muoki R, Mupura H, Murerwa E, Murisi C, Muroyiwa L, Muruvi M, Musemwa N, Mushure C, Mutero J, Mutero P, Mutumbu P, Mutya C, Muzanango L, Muzembi M, Muzungunye D, Mwazha V, Ncube T, Ndava T, Ndlovu N, Nehowa P, Ngara D, Nguruve L, Nhigo P, Nkiwane S, Nyanyai L, Nzombe J, Office E, Paul B, Pavari S, Ranganai S, Ratisai S, Rugara M, Rusere P, Sakala J, Sango P, Shava S, Shekede M, Shizha C, Sibanda T, Tapambwa N, Tembo J, Tinago N, Tinago V, Toindepi T, Tovigepi J, Tuhwe M, Tumbo K, Zaranyika T, Zaru T, Zimidzi K, Zindo M, Zindonda M, Zinhumwe N, Zishiri L, Ziyambi E, Zvinowanda J, Bepete E, Chiwira C, Chuma N, Fari A, Gavi S, Gunha V, Hakunandava F, Huku C, Hungwe G, Maduke G, Manyewe E, Mapfumo T, Marufu I, Mashiri C, Mazenge S, Mbinda E, Mhuri A, Muguti C, Munemo L, Musindo L, Ngada L, Nyembe D, Taruvinga R, Tobaiwa E, Banda S, Chaipa J, Chakaza P, Chandigere M, Changunduma A, Chibi C, Chidyagwai O, Chidza E, Chigatse N, Chikoto L, Chingware V, Chinhamo J, Chinhoro M, Chiripamberi A, Chitavati E, Chitiga R, Chivanga N, Chivese T, Chizema F, Dera S, Dhliwayo A, Dhononga P, Dimingo E, Dziyani M, Fambi T, Gambagamba L, Gandiyari S, Gomo C, Gore S, Gundani J, Gundani R, Gwarima L, Gwaringa C, Gwenya S, Hamilton R, Hlabano A, Hofisi E, Hofisi F, Hungwe S, Hwacha S, Hwara A, Jogwe R, Kanikani A, Kuchicha L, Kutsira M, Kuziyamisa K, Kuziyamisa M, Kwangware B, Lozani P, Mabuto J, Mabuto V, Mabvurwa L, Machacha R, Machaya C, Madembo R, Madya S, Madzingira S, Mafa L, Mafuta F, Mafuta J, Mahara A, Mahonye S, Maisva A, Makara A, Makover M, Mambongo E, Mambure M, Mandizvidza E, Mangena G, Manjengwa E, Manomano J, Mapfumo M, Mapfurire A, Maphosa L, Mapundo J, Mare D, Marecha F, Marecha S, Mashiri C, Masiya M, Masuku T, Masvimbo P, Matambo S, Matarise G, Matinanga L, Matizanadzo J, Maunganidze M, Mawere B, Mawire C, Mazvanya Y, Mbasera M, Mbono M, Mhakayakora C, Mhlanga N, Mhosva B, Moyo N, Moyo O, Moyo R, Mpakami C, Mpedzisi R, Mpofu E, Mpofu E, Mtetwa M, Muchakachi J, Mudadada T, Mudzingwa K, Mugwira M, Mukarati T, Munana A, Munazo J, Munyeki O, Mupfeka P, Murangandi G, Muranganwa M, Murenjekwa J, Muringo N, Mushaninga T, Mutaja F, Mutanha D, Mutemeri P, Mutero B, Muteya E, Muvembi S, Muzenda T, Mwenjota A, Ncube S, Ndabambi T, Ndava N, Ndlovu E, Nene E, Ngazimbi E, Ngwalati A, Nyama T, Nzembe A, Pabwaungana E, Phiri S, Pukuta R, Rambanapasi M, Rera T, Samanga V, Shirichena S, Shoko C, Shonhe M, Shuro C, Sibanda J, Sibangani E, Sibangani N, Sibindi N, Sitotombe M, Siwawa P, Tagwirei M, Taruvinga P, Tavagwisa A, Tete E, Tete Y, Thandiwe E, Tibugari A, Timothy S, Tongogara R, Tshuma L, Tsikira M, Tumba C, Watinaye R, Zhiradzango E, Zimunya E, Zinengwa L, Ziupfu M, Ziyambe J, Church JA, Desai A, Fundira D, Gough E, Kambarami RA, Matare CR, Malaba TR, Mupfudze T, Ngure F, Smith LE, Curtis V, Dickin KL, Habicht JP, Masimirembwa C, Morgan P, Pelto GH, Sheffner-Rogers C, Thelingwani R, Turner P, Zungu L, Makadzange T, Mujuru HA, Nyachowe C, Chakadai R, Chanyau G, Makamure MG, Chiwariro H, Mtetwa T, Chikunya J, Maguwu L, Nyadundu S, Moyo T, Chayima B, Mvindi L, Rwenhamo P, Muzvarwandoga S, Chimukangara R, Njovo H, Makoni T (2019). Independent and combined effects of improved water, sanitation, and hygiene, and improved complementary feeding, on child stunting and anaemia in rural Zimbabwe: a cluster-randomised trial. Lancet Glob Health.

[44] Rogawski McQuade ET, Platts-Mills JA, Gratz J, Zhang J, Moulton LH, Mutasa K (2020). Impact of water quality, sanitation, handwashing, and nutritional interventions on enteric infections in rural Zimbabwe: the sanitation hygiene infant nutrition efficacy (SHINE) trial. J Infect Dis.

[45] Feed the Future Innovation Lab for Livestock Systems. CAGED: Campylobacter genomics and environmental enteric dysfunction. http://livestocklab.ifas.ufl.edu/projects/caged-project/. Accessed 15 Jul 2019.

[46] Oberhelman RA, Gilman RH, Sheen P, Cordova J, Zimic M, Cabrera L, et al. An intervention-control study of corralling of free-ranging chickens to control Campylobacter infections among children in a Peruvian periurban shantytown. Am J Tropical Med Hygiene. 2006;74(6):1054-9. 16760519

[47] Prendergast AJ, Gharpure R, Mor S, Viney M, Dube K, Lello J, Berger C, Siwila J, Joyeux M, Hodobo T, Hurt L, Brown T, Hoto P, Tavengwa N, Mutasa K, Craddock S, Chasekwa B, Robertson RC, Evans C, Chidhanguro D, Mutasa B, Majo F, Smith LE, Hirai M, Ntozini R, Humphrey JH, Berendes D (2019). Putting the “a” into WaSH: a call for integrated management of water, animals, sanitation, and hygiene. The Lancet Planetary Health.

[48] Zinsstag J, Schelling E, Waltner-Toews D, Tanner M (2011). From “one medicine” to “one health” and systemic approaches to health and well-being. Preventive Veterinary Med.

