# Supplementary material for: The role of chicken management practices in children’s exposure to environmental contamination: a mixed-methods analysis
Source: BMC Public Health. 2021 Jun 8;21:1097. doi: 10.1186/s12889-021-11025-y (PMC8188703; doi:10.1186/s12889-021-11025-y)
Supplement: Supplementary file 1 — Additional file 1: Supplement 1. Semi-Structured Interview Guide: In-Depth Interviews with Women. Supplemental file containing the full semi-structured interview guide for the in-depth interviews with women during the midline evaluation. This interview guide was developed by the authors of this manuscript for the purposes of this study. [file 12889_2021_11025_MOESM1_ESM.docx]

# Supplement 1: Semi-Structured Interview Guide: In-Depth Interviews with Women

*To be conducted at the end of Day 2 of observation with the primary caregiver of the index child (woman). Interviews must be conducted in private, outside of the hearing range of other adults and family members, except young children.*

**Structured Questions**

- 1. How many chickens does your household have?
  2. *If ACGG* *Household:*
     1. How many chickens did your household receive?
     2. When did the chickens arrive?
     3. How many of the ACGG chickens do you still have?
  3. How many hours per day do you usually spend caring for chickens?
  4. Does anyone else help you with you the chicken production activities?
     1. If so, who?
     2. How much of the work do you yourself do?
     3. Do you compensate the person who helps you (money, labor, eggs, etc)

**Semi Structured Questions**

- 1. Where do the chickens go at night? Why here?
  2. Where do the other livestock go at night? Why here? (goat, cattle, donkey)
  3. Which factors do you think are most important for your own nutrition (or ‘healthy’)? (frequency, portion size, allocation, etc.)
  4. Which factors do you think are most important for your child’s nutrition? (frequency, portion size, allocation, etc.)
  5. Do you believe that chickens will help your child’s nutrition?
     1. If so, in what way or ways?
  6. What are the main challenges to chicken production?
  7. Are you satisfied with your access to sanitation and hygiene, such as waste disposal, latrines, and hand washing? If yes, why? If no, why not?
  8. What do you think is the role of sanitation and hygiene in your child’s nutrition?
  9. When do you perceive that a child should wash his/her hands? [perception of dirty]
  10. Do you think it is important for chickens to roam freely or to be cooped up? Why?
  11. Do you ever try to corral chickens? Do you ever try to keep your children from them? Why or why not?
  12. How do you feel that chickens contribute to the sanitation and hygiene of your household?
      1. For ACGG: how do you feel that sanitation and hygiene conditions have changed since you received the new chickens? What are the benefits/challenges?
  13. What would be most useful to you to improve nutrition of your household?
  14. What would be most useful to you for improving the nutritional status of [index child]?
