# Supplementary material for: The role of chicken management practices in children’s exposure to environmental contamination: a mixed-methods analysis
Source: BMC Public Health. 2021 Jun 8;21:1097. doi: 10.1186/s12889-021-11025-y (PMC8188703; doi:10.1186/s12889-021-11025-y)
Supplement: Supplementary file 2 — Additional file 1: Supplement 2. Direct Household Observation Questionnaire. This file contains the full data collection instrument used for the direct observations of the 18 households included in the midline observation phase. [file 12889_2021_11025_MOESM2_ESM.docx]

# Supplement 2: Direct Household Observation Questionnaire

# Direct Observation: Day 1

# Section 1: Survey Information

| **Question #** | **Question** | **Code** |  |
| --- | --- | --- | --- |
|  | Date (DD/MM/YYYY) |  | \|  \|  \|  \|  \|  \|  \|  \|  \| \| --- \| --- \| --- \| --- \| --- \| --- \| --- \| --- \| |
|  | Time (12 hour AM/PM) | (HH:MM) | \|  \|  \|  \|  \| A/P \| M \| \| --- \| --- \| --- \| --- \| --- \| --- \| |
|  | Supervisor ID |  | \|  \|  \| \| --- \| --- \| |
|  | Supervisor Name |  |  |
|  | Research Assistant ID |  | \|  \|  \| \| --- \| --- \| |
|  | Research Assistant Name |  |  |
|  | Enumerator ID |  | \|  \|  \| \| --- \| --- \| |
|  | Enumerator Name |  |  |
|  | Region | 1. **Amhara** 2. **Oromia** |  |
|  | Kebele | 1. Guta (control) 2. Dikuli 3. Ashuda 4. DembiGobu (control) 5. OdaHaro 6. Tarkanfate Gibe |  |
|  | Treatment status of kebele | 1. **ACGG** 2. **ATONU** 3. **Control** |  |
|  | Household ID | **Two-Digit Number** |  |

## Section 2: Basic Information

*Ask the woman respondent the following questions:*

| **Question #** | **Question** | **Code** | **Response** | **Skip** |
| --- | --- | --- | --- | --- |
|  | Name of woman respondent |  |  |  |
|  | Age of woman respondent |  |  |  |
|  | Name of index child |  |  |  |
|  | Sex of index child | 1. Male 2. Female |  |  |
|  | Birth Date of Index Child *(from health card)* | DD/MM/YYYY | \|  \|  \|  \|  \|  \|  \|  \|  \| \| --- \| --- \| --- \| --- \| --- \| --- \| --- \| --- \| | *If child less than 6 months or over 36 months of age, end interview* |

## Section 3: Basic Household Information, observed

***The following questions are for OBSERVATION ONLY. These questions are asked only on the 1st day of observation.***

1. Is there a latrine for the household? (RA will ask “Can I use the latrine Mam?”)
   1. Household has own latrine
   2. Household uses neighbor’s latrine
   3. Household uses public/ shared latrine
   4. Household does not use latrine>>*Skip to Q5*

1. Does the latrine have a vent pipe?
2. Yes
3. No>>*Skip to Q3*

2a) Does the ventilation work?

1. Yes
2. No
3. Is the latrine overflowing?
4. Yes
5. No
6. Evidence of use (trodden path test)? Explain the trodden path test
7. Yes
8. No
9. Is there a hand washing station? Many of the households just have one so we just ask about the one hand washing station or the one closest to the bathroom?
10. Yes
11. No
12. Is there soap near hand washing station?
13. Yes
14. No
15. What is the source of water most commonly used for drinking? (RA will ask for “drinking water”, and then ask about where they get it from)
16. Standpipe/borehole on property
17. Standpipe/borehole not on property
18. Running water
19. Surface water on property
20. Surface water not on property
21. Filtered water system in household
22. *(Ask where applicable)* If water is not on property, how far does the household usually walk ONE WAY for drinking water? If the mother and child go get water – do we follow? We said RAs should follow if its less than 30 mins

__ __ __ minutes

1. What kind of floor material does the household (main living room) have indoors?
2. Dung
3. Dirt
4. Smooth concrete
5. Unsmooth concrete
6. Some areas are concrete, some are dirt/dung
7. Floor covered with plastic/_______
8. Other:___________
9. Where is most of the cooking done?
10. Inside the main household
11. Separate room outside the household
12. Outside, out in the open
13. **Drinking water storage containers: If there are a lot of jerry cans, write down for number and proportion that were covered. For example, if 3/6 cans were covered, you can say partial and write down the details.**

| **Drinking water storage container** | **Covered?** | | |
| --- | --- | --- | --- |
|  | **Not** | **Partially** | **Completely** |
| Drum | \|  \| \| --- \| | \|  \| \| --- \| | \|  \| \| --- \| |
| 20-25l Plastic container (“jerry can”/ narrow mouth) | \|  \| \| --- \| | \|  \| \| --- \| | \|  \| \| --- \| |
| Bucket | \|  \| \| --- \| | \|  \| \| --- \| | \|  \| \| --- \| |
| Clay pot | \|  \| \| --- \| | \|  \| \| --- \| | \|  \| \| --- \| |
| Other __________________ | \|  \| \| --- \| | \|  \| \| --- \| | \|  \| \| --- \| |

1. *(Ask where applicable)* What do you use to scoop drinking water from the bucket/ drum/ clay pot (Tick all that apply)?
2. Specific scooping cup
3. Specific ladle with long handle
4. Any cup
5. Any container
6. Other, specify (_________________________)
7. Does the household have a chicken coop?
8. Yes
9. No >>Q15
10. Take (a) picture(s) of the fencing, coop structures for chickens.
11. Was/were picture/s taken?
12. Yes
13. No (specify)__________________
14. Are there fences/structures for any other animals?
15. Yes
16. No >>*next section*
17. Take (a) picture(s) of the fencing or structures for animals other than chickens.
18. Was/were picture/s taken?
19. Yes

No (specify)__________________

# Section 4: Household Characteristics Observed, Daily

***The following questions are asked at the start of each day of observation:***

1. Are chicken feces visible in the yard?
2. Yes
3. No
4. Are other animal feces visible in the yard?

1.Yes

2. No

1. Are human feces visible in the yard?

1.Yes

2. No

1. Is there evidence of a recent hand washing event (Tick all that apply)?
2. Yes, water on the ground next to handwashing station
3. Yes, container/tippy tap with water
4. No clear evidence
5. Couldn’t tell because it was a rainy day
6. Other, specify (_________________________)
7. Where does the child play?
8. Inside the house
9. Outside the house

22a) ~~I~~Describe the play surface: (mat, blanket, bed (traditional raised up or modern), pillows).

(Write observations upon arrival if the child is in the play surface; if the child the child is sleeping, and wakes up later to play, describe the play area)

22b~~) If “Yes,~~ is the play surface clean? (RAs can write “ I think this is dirty because of x x x”)

1. Yes (animal feces, dirt or leftover food, child feces, flies)
2. No
3. Cannot see

# Section 5: Hourly Observations

***Please complete this list of observations at the start of every hour, on the hour, from when you arrive to when you leave.***

|  | On arrival | At 1 hours | At 2 hours | At 3 hours |
| --- | --- | --- | --- | --- |
| Time *(use military time) Planned*  ***Actual*** | __ : __  __ : __ | __ : __  __ : __ | __ : __  __ : __ | __ : __  __ : __ |
| 1. Mother’s hands visibly clean | 1 Yes 2 No 3 Cannot see | 1 Yes 2 No 3 Cannot see | 1 Yes 2 No 3 Cannot see | **1 Yes 2 No**  3 Cannot see |
| 1. Baby’s hands visibly clean | 1 Yes 2 No 3 Cannot see | 1 Yes 2 No 3 Cannot see | 1 Yes 2 No 3 Cannot see | **1 Yes 2 No**  3 Cannot see |
| 1. Diaper or child’s bottom is clean | 1 Yes 2 No 3 Cannot see | 1 Yes 2 No 3 Cannot see | 1 Yes 2 No 3 Cannot see | **1 Yes 2 No**  3 Cannot see |
| 1. There is stagnant water visible outside the house | 1 Yes 2 No 3 Cannot see | 1 Yes 2 No 3 Cannot see | 1 Yes 2 No 3 Cannot see | **1 Yes 2 No**  3 Cannot see |
| 1. There are unwashed utensils | 1 Yes 2 No 3 Cannot see | 1 Yes 2 No 3 Cannot see | 1 Yes 2 No 3 Cannot see | **1 Yes 2 No**  3 Cannot see |
| 1. There is uncovered food (after the meal is completed) | 1 Yes 2 No 3 Cannot see | 1 Yes 2 No 3 Cannot see | 1 Yes 2 No 3 Cannot see | **1 Yes 2 No**  3 Cannot see |
| 1. ~~Spill on kitchen floor (food or drink)~~   Kitchen is visibly dirty when its not being used | ~~1 Yes 2 No~~ ~~3 Cannot see~~ | ~~1 Yes 2 No~~ ~~3 Cannot see~~ | ~~1 Yes 2 No~~ ~~3 Cannot see~~ | **~~1 Yes 2 No~~**  ~~3 Cannot see~~ |
| 1. There are poultry feces visible on kitchen floor | 1 Yes 2 No 3 Cannot see | 1 Yes 2 No 3 Cannot see | 1 Yes 2 No 3 Cannot see | **1 Yes 2 No**  3 Cannot see |
| 1. There are animals inside the house. | 1 Yes 2 No 3 Cannot see | 1 Yes 2 No 3 Cannot see | 1 Yes 2 No 3 Cannot see | **1 Yes 2 No**   1. Cannot see |
| 9a) If “Yes” for 9, list animal types and numbers, e.g. 2 goats, 3 chickens, cats, or dogs, |  |  |  |  |
| 1. Kitchen yard is swept or cleaned | 1 Yes 2 No 3 Cannot see | 1 Yes 2 No 3 Cannot see | 1 Yes 2 No 3 Cannot see | **1 Yes 2 No**  3 Cannot see |
| 1. Where is child at this time? Circle all the applies and put the most common | 1. Dirt/dung floor inside **2. Finished floor inside**  **3. Dirt/dung floor outside**  **4. Finished floor outside**  **5. On mother (hip)**  **6. On mother (lap)**  **7. On mother (back)**  **8. On mat or blanket**  **9. Other(specify)** | 1. Dirt/dung floor inside **2. Finished floor inside**  **3. Dirt/dung floor outside**  **4. Finished floor outside**  **5. On mother (hip)**  **6. On mother (lap)**  **7. On mother (back)**  **8. On mat or blanket**  **9. Other(specify)** | 1. Dirt/dung floor inside **2. Finished floor inside**  **3. Dirt/dung floor outside**  **4. Finished floor outside**  **5. On mother (hip)**  **6. On mother (lap)**  **7. On mother (back)**  **8. On mat or blanket**  **9. Other(specify)** | 1. Dirt/dung floor inside **2. Finished floor inside**  **3. Dirt/dung floor outside**  **4. Finished floor outside**  **5. On mother (hip)**  **6. On mother (lap)**  **7. On mother (back)**  **8. On mat or blanket**  **9. Other(specify)** |
| 1. How many chickens are within 5 meters (5 walks) of the child, at any time within the hour (try to time if you can)? | __ __ chickens | __ __ chickens | __ __ chickens | __ __ chickens |
| 1. Are feces visible within 5 meters (5 walks) of the child? | 1 Yes 2 No3 Cannot see | 1 Yes 2 No3 Cannot see | 1 Yes 2 No3 Cannot see | **1 Yes 2 No**  **3 Cannot see** |
| 1. Is the area where the child is playing swept/clean? | 1 Yes 2 No3 Cannot see4. Not applicable | 1 Yes 2 No3 Cannot see4. Not applicable | 1 Yes 2 No3 Cannot see4. Not applicable | 1 Yes 2 No3 Cannot see **4. Not applicable** |
| 1. A hand washing event |  |  |  |  |
| ***Motor development:***   1. Observe and check all that apply, | Laying, no sitting or movement  sit with support  sitting without support  creep on stomach  hands-and-knees crawling  standing with assistance  walking with assistance  standing alone  walking alone  run  other (please note) | Laying, no sitting or movement  sit with support  sitting without support  creep on stomach  hands-and-knees crawling  standing with assistance  walking with assistance  standing alone  walking alone  run  other (please note) | Laying, no sitting or movement  sit with support  sitting without support  creep on stomach  hands-and-knees crawling  standing with assistance  walking with assistance  standing alone  walking alone  run  other (please note) | Laying, no sitting or movement  sit with support  sitting without support  creep on stomach  hands-and-knees crawling  standing with assistance  walking with assistance  standing alone  walking alone  run  other (please note) |
| ***Behavior & health***  Observe and *check all that apply* | Infant appears healthy  *Infant is alert and active when awake, is feeding well, and can be comforted when crying*  Infant is lethargic  *Has little or no energy, drowsy or sluggish, difficult to wake for feedings, Not alert or attentive to sounds and visual stimulation*  Infant is persistently crying or irritable  *Continuously fretful and fussy, Cries for long periods or very suddenly, Has a cry that sounds unusual*  Infant seems sick  *Is pale or flushed, has problems breathing, is vomiting or has diarrhea*  other (please note): | Infant appears healthy  *Infant is alert and active when awake, is feeding well, and can be comforted when crying*  Infant is lethargic  *Has little or no energy, drowsy or sluggish, difficult to wake for feedings, Not alert or attentive to sounds and visual stimulation*  Infant is persistently crying or irritable  *Continuously fretful and fussy, Cries for long periods or very suddenly, Has a cry that sounds unusual*  Infant seems sick  *Is pale or flushed, has problems breathing, is vomiting or has diarrhea*  other (please note): | Infant appears healthy  *Infant is alert and active when awake, is feeding well, and can be comforted when crying*  Infant is lethargic  *Has little or no energy, drowsy or sluggish, difficult to wake for feedings, Not alert or attentive to sounds and visual stimulation*  Infant is persistently crying or irritable  *Continuously fretful and fussy, Cries for long periods or very suddenly, Has a cry that sounds unusual*  Infant seems sick  *Is pale or flushed, has problems breathing, is vomiting or has diarrhea*  other (please note): | Infant appears healthy  *Infant is alert and active when awake, is feeding well, and can be comforted when crying*  Infant is lethargic  *Has little or no energy, drowsy or sluggish, difficult to wake for feedings, Not alert or attentive to sounds and visual stimulation*  Infant is persistently crying or irritable  *Continuously fretful and fussy, Cries for long periods or very suddenly, Has a cry that sounds unusual*  Infant seems sick  *Is pale or flushed, has problems breathing, is vomiting or has diarrhea*  other (please note): |

**15. Number of any animals (regardless of the ownership) present in the yard at the time of observation**

|  | **On Arrival** | | | **At 1 hours** | | | **At 2 hours** | | | **At 3 hours** | | |
| --- | --- | --- | --- | --- | --- | --- | --- | --- | --- | --- | --- | --- |
| **TIME** |  | | |  | | |  | | |  | | |
| **Domestic Animals** | Corralled | Not Corralled | Corralled | | Not Corralled | Corralled | | Not Corralled | Corralled | | Not Corralled |  |
| **Cattle** |  |  |  | |  |  | |  |  | |  |  |
| **Goats** |  |  |  | |  |  | |  |  | |  |  |
| **Chickens** |  |  |  | |  |  | |  |  | |  |  |
| **Mules/Donkeys/horses** |  |  |  | |  |  | |  |  | |  |  |
| **Other:** *write in all other animals (cats/ dogs):*  *____________________________________* |  |  |  | |  |  | |  |  | |  |  |

# Section 6: Child Sanitation Practices (Defecation/kaka/poop only, not urine); if the child did not defecate, put did not defecate (NA) and if the child did defecate but there’s no clean up, put did not clean up

| ***Record first three nappy changes after defecation per visit.*** | **Defecation 1** | **Defecation 2** | **Defecation 3** |
| --- | --- | --- | --- |
| Time *(use military time. If no nappy changes occur, write 99:99)* | __ : __ | __ : __ | __ : __ |
| 1. Who initiated cleaning the child  (Enter: 1=child indicated need; 2=caregiver; 3 = others in the household; 4= did not clean up9=not able to observe) |  |  |  |
| 2. What was done with the fecal material  (Enter: 1=disposed of via toilet/latrine; 2=wrapped up for disposal in garbage; 3=buried; 4=tossed in yard; 5=left untended for > 30 min; 6= other specify 9=not able to observe) |  |  |  |
| 3. How was the child’s bottom cleaned?  (Enter: 1=soap and water; 2=rinsed only with water; 3=wiped with cloth only; 4=Wiped with paper; 9=not able to observe) |  |  |  |
| 4. For children with nappies, did caregiver wash his/her hands within 5 min after cleaning the child’s bottom (nappy change?  (Enter: 0 = none/no: 1=one hand; 2=two hands;3= water only/no rubbing agent;4= Soap; 5= ash; 6=other; 7=run to waste water; 8= still water; 9=unable to observe) |  |  |  |
| 5. For children without nappies, did caregiver wash his/her hands within 5 min after assisting a toddler to clean up after defecation?  (Enter: 0 = none/no: 1=one hand; 2=two hands;3= water only/no rubbing agent;4= Soap; 5= ash; 6=other; 7=run to waste water; 8= still water; 9=unable to observe) |  |  |  |

# Section 7: Behavior checklist

**Add a tally in the appropriate box for the given hour, any time each of these behaviors is observed.**

|  | Hour 0-1 | Hour 1-2 | Hour 2-3 |  | |
| --- | --- | --- | --- | --- | --- |
| ***TIME: FILL IN WITH HOURS OF OBSERVATION*** | __ : __  to  __ : __ | __ : __  to  __ : __ | __ : __  to  __ : __ |  |  |
| **Behavior** | **Number of Times Behavior was Observed in this Hour Block (TALLY)** | | | |  |
| Baby put fingers in mouth |  |  |  |  | |
| Baby put dirt in mouth |  |  |  |  |  |
| Baby put visibly dirty object or food (dirty food is defined as something dropped on the floor and consumed by the child) in mouth |  |  |  |  |  |
| Baby put not visibly dirty object in mouth |  |  |  |  |  |
| Baby touched an animal |  |  |  |  |  |
| Baby ate freshly cooked food |  |  |  |  |  |
| Baby ate leftover food that was covered |  |  |  |  |  |
| Baby ate leftover food that was not covered |  |  |  |  |  |
| Baby drank water |  |  |  |  |  |
| Baby’s hands were washed with soap/ cleaning agent |  |  |  |  |  |
| Baby’s hands were washed without soap/ cleaning agent |  |  |  |  |  |
| Mother’s hands were washed with soap/ cleaning agent |  |  |  |  | |
| Mother’s hands were washed without soap/ cleaning agent |  |  |  |  | |
| Other behaviors? |  |  |  |  | |

# Section 8: Researcher Field Notes:

*Please describe any other details of behaviors observed during your observation today, interactions or other interesting or relevant information.*

# Direct Observation: Day 2

# Section 8: Survey Information

| **Question #** | **Question** | **Code** |  |
| --- | --- | --- | --- |
|  | Date (DD/MM/YYYY) |  | \|  \|  \|  \|  \|  \|  \|  \|  \| \| --- \| --- \| --- \| --- \| --- \| --- \| --- \| --- \| |
|  | Supervisor ID |  | \|  \|  \| \| --- \| --- \| |
|  | Supervisor Name |  |  |
|  | Research Assistant ID |  | \|  \|  \| \| --- \| --- \| |
|  | Research Assistant Name |  |  |
|  | Enumerator ID |  | \|  \|  \| \| --- \| --- \| |
|  | Enumerator Name |  |  |
|  | Region | 1. **Amhara** 2. **Oromia** |  |
|  | Kebele | 1. **1** 2. **2** 3. **3** 4. **4** 5. **5** 6. **6** |  |
|  | Treatment status of kebele | 1. **ACGG** 2. **ATONU** 3. **Control** |  |
|  | Household ID | **Two-Digit Number** |  |

# Section 9: Daily Observed Household Characteristics

***The following questions are asked at the start of each day of observation:***

1. Are chicken feces visible in the yard?
2. Yes
3. No
4. Are other animal feces visible in the yard?

1.Yes

2. No

1. Are human feces visible in the yard?

1.Yes

2. No

1. Is there evidence of a recent hand washing event (Tick all that apply)?
2. Yes, water on the ground next to handwashing station
3. Yes, container/tippy tap with water
4. No clear evidence
5. Couldn’t tell because it was a rainy day
6. Other, specify (_________________________)
7. Child has specific play surface
8. Yes
9. No
10. Cannot see

5a) If “Yes” for 5, describe the play surface:

5b) If “Yes” for 5, is the play surface clean?

1. Yes
2. No
3. Cannot see

|  | On arrival | At 1 hours | At 2 hours | At 3 hours |
| --- | --- | --- | --- | --- |
| Time *(use military time) Planned*  ***Actual*** | __ : __  __ : __ | __ : __  __ : __ | __ : __  __ : __ | __ : __  __ : __ |
| 1. Mother’s hands visibly clean | 1 Yes 2 No 3 Cannot see | 1 Yes 2 No 3 Cannot see | 1 Yes 2 No 3 Cannot see | **1 Yes 2 No**  3 Cannot see |
| 1. Baby’s hands visibly clean | 1 Yes 2 No 3 Cannot see | 1 Yes 2 No 3 Cannot see | 1 Yes 2 No 3 Cannot see | **1 Yes 2 No**  3 Cannot see |
| 1. Diaper or child’s bottom is clean | 1 Yes 2 No 3 Cannot see | 1 Yes 2 No 3 Cannot see | 1 Yes 2 No 3 Cannot see | **1 Yes 2 No**  3 Cannot see |
| 1. There is stagnant water visible outside the house | 1 Yes 2 No 3 Cannot see | 1 Yes 2 No 3 Cannot see | 1 Yes 2 No 3 Cannot see | **1 Yes 2 No**  3 Cannot see |
| 1. There are unwashed utensils | 1 Yes 2 No 3 Cannot see | 1 Yes 2 No 3 Cannot see | 1 Yes 2 No 3 Cannot see | **1 Yes 2 No**  3 Cannot see |
| 1. There is uncovered food (after the meal is completed) | 1 Yes 2 No 3 Cannot see | 1 Yes 2 No 3 Cannot see | 1 Yes 2 No 3 Cannot see | **1 Yes 2 No**  3 Cannot see |
| 1. ~~Spill on kitchen floor (food or drink)~~   Kitchen is visibly dirty when its not being used | ~~1 Yes 2 No~~ ~~3 Cannot see~~ | ~~1 Yes 2 No~~ ~~3 Cannot see~~ | ~~1 Yes 2 No~~ ~~3 Cannot see~~ | **~~1 Yes 2 No~~**  ~~3 Cannot see~~ |
| 1. There are poultry feces visible on kitchen floor | 1 Yes 2 No 3 Cannot see | 1 Yes 2 No 3 Cannot see | 1 Yes 2 No 3 Cannot see | **1 Yes 2 No**  3 Cannot see |
| 1. There are animals inside the house. | 1 Yes 2 No 3 Cannot see | 1 Yes 2 No 3 Cannot see | 1 Yes 2 No 3 Cannot see | **1 Yes 2 No**   1. Cannot see |
| 9a) If “Yes” for 9, list animal types and numbers, e.g. 2 goats, 3 chickens, cats, or dogs, |  |  |  |  |
| 1. Kitchen yard is swept or cleaned | 1 Yes 2 No 3 Cannot see | 1 Yes 2 No 3 Cannot see | 1 Yes 2 No 3 Cannot see | **1 Yes 2 No**  3 Cannot see |
| 1. Where is child at this time? Circle all the applies and put the most common | 1. Dirt/dung floor inside **2. Finished floor inside**  **3. Dirt/dung floor outside**  **4. Finished floor outside**  **5. On mother (hip)**  **6. On mother (lap)**  **7. On mother (back)**  **8. On mat or blanket**  **9. Other(specify)** | 1. Dirt/dung floor inside **2. Finished floor inside**  **3. Dirt/dung floor outside**  **4. Finished floor outside**  **5. On mother (hip)**  **6. On mother (lap)**  **7. On mother (back)**  **8. On mat or blanket**  **9. Other(specify)** | 1. Dirt/dung floor inside **2. Finished floor inside**  **3. Dirt/dung floor outside**  **4. Finished floor outside**  **5. On mother (hip)**  **6. On mother (lap)**  **7. On mother (back)**  **8. On mat or blanket**  **9. Other(specify)** | 1. Dirt/dung floor inside **2. Finished floor inside**  **3. Dirt/dung floor outside**  **4. Finished floor outside**  **5. On mother (hip)**  **6. On mother (lap)**  **7. On mother (back)**  **8. On mat or blanket**  **9. Other(specify)** |
| 1. How many chickens are within 5 meters (5 walks) of the child? | __ __ chickens | __ __ chickens | __ __ chickens | __ __ chickens |
| 1. Are feces visible within 5 meters (5 walks of the child? | 1 Yes 2 No3 Cannot see | 1 Yes 2 No3 Cannot see | 1 Yes 2 No3 Cannot see | **1 Yes 2 No**  **3 Cannot see** |
| 1. Is the area where the child is playing swept/clean? | 1 Yes 2 No3 Cannot see4. Not applicable | 1 Yes 2 No3 Cannot see4. Not applicable | 1 Yes 2 No3 Cannot see4. Not applicable | 1 Yes 2 No3 Cannot see **4. Not applicable** |
| 1. A hand washing event |  |  |  |  |
| ***Motor development:***   1. Observe and check all that apply, | Laying, no sitting or movement  sit with support  sitting without support  creep on stomach  hands-and-knees crawling  standing with assistance  walking with assistance  standing alone  walking alone  run  other (please note) | Laying, no sitting or movement  sit with support  sitting without support  creep on stomach  hands-and-knees crawling  standing with assistance  walking with assistance  standing alone  walking alone  run  other (please note) | Laying, no sitting or movement  sit with support  sitting without support  creep on stomach  hands-and-knees crawling  standing with assistance  walking with assistance  standing alone  walking alone  run  other (please note) | Laying, no sitting or movement  sit with support  sitting without support  creep on stomach  hands-and-knees crawling  standing with assistance  walking with assistance  standing alone  walking alone  run  other (please note) |
| ***Behavior & health***  Observe and *check all that apply* | Infant appears healthy  *Infant is alert and active when awake, is feeding well, and can be comforted when crying*  Infant is lethargic  *Has little or no energy, drowsy or sluggish, difficult to wake for feedings, Not alert or attentive to sounds and visual stimulation*  Infant is persistently crying or irritable  *Continuously fretful and fussy, Cries for long periods or very suddenly, Has a cry that sounds unusual*  Infant seems sick  *Is pale or flushed, has problems breathing, is vomiting or has diarrhea*  other (please note): | Infant appears healthy  *Infant is alert and active when awake, is feeding well, and can be comforted when crying*  Infant is lethargic  *Has little or no energy, drowsy or sluggish, difficult to wake for feedings, Not alert or attentive to sounds and visual stimulation*  Infant is persistently crying or irritable  *Continuously fretful and fussy, Cries for long periods or very suddenly, Has a cry that sounds unusual*  Infant seems sick  *Is pale or flushed, has problems breathing, is vomiting or has diarrhea*  other (please note): | Infant appears healthy  *Infant is alert and active when awake, is feeding well, and can be comforted when crying*  Infant is lethargic  *Has little or no energy, drowsy or sluggish, difficult to wake for feedings, Not alert or attentive to sounds and visual stimulation*  Infant is persistently crying or irritable  *Continuously fretful and fussy, Cries for long periods or very suddenly, Has a cry that sounds unusual*  Infant seems sick  *Is pale or flushed, has problems breathing, is vomiting or has diarrhea*  other (please note): | Infant appears healthy  *Infant is alert and active when awake, is feeding well, and can be comforted when crying*  Infant is lethargic  *Has little or no energy, drowsy or sluggish, difficult to wake for feedings, Not alert or attentive to sounds and visual stimulation*  Infant is persistently crying or irritable  *Continuously fretful and fussy, Cries for long periods or very suddenly, Has a cry that sounds unusual*  Infant seems sick  *Is pale or flushed, has problems breathing, is vomiting or has diarrhea*  other (please note): |

# Section 6: Child Sanitation Practices (Defecation/kaka/poop only, not urine); if the child did not defecate, put did not defcate (NA) and if the child did defecate but there’s no clean up, put did not clean up

| ***Record first three nappy changes after defecation per visit.*** | **Defecation 1** | **Defecation 2** | **Defecation 3** |
| --- | --- | --- | --- |
| Time *(use military time. If no nappy changes occur, write 99:99)* | __ : __ | __ : __ | __ : __ |
| 1. Who initiated cleaning the child  (Enter: 1=child indicated need; 2=caregiver; 3 = others in the household; 4= did not clean up9=not able to observe) |  |  |  |
| 2. What was done with the fecal material  (Enter: 1=disposed of via toilet/latrine; 2=wrapped up for disposal in garbage; 3=buried; 4=tossed in yard; 5=left untended for > 30 min; 6= other specify 9=not able to observe) |  |  |  |
| 3. How was the child’s bottom cleaned?  (Enter: 1=soap and water; 2=rinsed only with water; 3=wiped with cloth only; 4=Wiped with paper; 9=not able to observe) |  |  |  |
| 4. Did caregiver wash his/her hands within 5 min after cleaning the child’s bottom (nappy change)?>>*Use Q5 if child does not use nappies*  (Enter: 0 = none/no: 1=one hand; 2=two hands;3= water only/no rubbing agent;4= Soap; 5= ash; 6=other; 7=run to waste water; 8= still water; 9=unable to observe) |  |  |  |
| 5. Did caregiver wash his/her hands within 5 min after assisting a toddler to clean up after defecation?  (Enter: 0 = none/no: 1=one hand; 2=two hands;3= water only/no rubbing agent;4= Soap; 5= ash; 6=other; 7=run to waste water; 8= still water; 9=unable to observe) |  |  |  |

# Section 7: Behavior checklist

**Add a tally in the appropriate box for the given hour, any time each of these behaviors is observed.**

|  | Hour 0-1 | Hour 1-2 | Hour 2-3 |  | |
| --- | --- | --- | --- | --- | --- |
| ***TIME: FILL IN WITH HOURS OF OBSERVATION*** | __ : __  to  __ : __ | __ : __  to  __ : __ | __ : __  to  __ : __ |  |  |
| **Behavior** | **Number of Times Behavior was Observed in this Hour Block (TALLY)** | | | |  |
| Baby put fingers in mouth |  |  |  |  | |
| Baby put dirt in mouth |  |  |  |  |  |
| Baby put visibly dirty object in mouth |  |  |  |  |  |
| Baby put not visibly dirty object in mouth |  |  |  |  |  |
| Baby touched an animal |  |  |  |  |  |
| Baby ate freshly cooked food |  |  |  |  |  |
| Baby ate leftover food that was covered |  |  |  |  |  |
| Baby ate leftover food that was not covered |  |  |  |  |  |
| Baby drank water |  |  |  |  |  |
| Baby’s hands were washed with soap/ cleaning agent |  |  |  |  |  |
| Baby’s hands were washed without soap/ cleaning agent |  |  |  |  |  |
| Mother’s hands were washed with soap/ cleaning agent |  |  |  |  | |
| Mother’s hands were washed without soap/ cleaning agent |  |  |  |  | |
| Other behaviors? |  |  |  |  | |

# Section 10: Researcher Field Notes:

*Please describe any other details of behaviors observed during your observation today, interactions or other interesting or relevant information.*
