# Supplementary material for: The role of chicken management practices in children’s exposure to environmental contamination: a mixed-methods analysis
Source: BMC Public Health. 2021 Jun 8;21:1097. doi: 10.1186/s12889-021-11025-y (PMC8188703; doi:10.1186/s12889-021-11025-y)
Supplement: Supplementary file 3 — Additional file 3: Supplement 3. Codebook for Women’s In-depth Interviews. This supplement contains the codebook with all codes used to analyze the transcripts of the in-depth interviews using thematic analysis. These codes were exported from NVivo after analysis. [file 12889_2021_11025_MOESM3_ESM.docx]

# Supplement 3: Codebook for Women’s In-depth Interviews^i^

| Name | Description | Files | References |
| --- | --- | --- | --- |
| Gender | Gender relations/dynamics intrahousehold or in the community | 9 | 15 |
| Decisions | Who makes decisions in the household | 3 | 4 |
| Roles | Describes certain activities to be "men's" or "women's" | 8 | 11 |
| Help | Help with household and farm/livestock work | 18 | 117 |
| Family | Other family members helping out | 4 | 7 |
| Husband | References specifically to the husband’s help and the nature of that help | 16 | 72 |
| Chickens | Husband helps with the chickens | 9 | 14 |
| Childcare | Husband holds the child, plays with the child, watches child, washes the child, takes the child to health appointments, takes care of the child when sick, feeds the child | 11 | 16 |
| Chores | Husband brings water, fire, washing clothes, or does other household chores | 4 | 7 |
| Expand | Husband or wife mention husband would do other tasks to expand his roles | 4 | 5 |
| None | Does not receive help from others | 9 | 14 |
| Older kids | Reference to older kids helping out | 11 | 19 |
| Management | Discussion of chicken management practices | 18 | 450 |
| Air | A reference to chickens needing air/space to run around | 7 | 9 |
| Conflict | Raising conflict (i.e. with neighbors) with respect to management | 2 | 3 |
| Dirty | Dirtiness as negative aspect of chickens | 13 | 24 |
| Unpleasant | Unpleasantness with respect to chicken management practices | 2 | 3 |
| Disease | Chicken death or disease as a challenge to raising chickens | 12 | 23 |
| Feed | How the requirement for feed influences management practices, or as a struggle for chicken production | 10 | 19 |
| Location | Location of poultry housing or where poultry hang out | 18 | 215 |
| Coop | Any text referring to a coop used for keeping poultry | 17 | 71 |
| Corral | Animal (chicken or livestock) corral | 11 | 16 |
| House | Animals being in the main house | 14 | 42 |
| Night | Nighttime, including where the chickens spend the night, roost, sleep | 18 | 44 |
| Roam | Chickens roaming | 17 | 32 |
| Meds | Vaccine or medicine as a challenge to chicken production | 6 | 7 |
| Mess up | Chickens messing up crops/storage/yard/neighbor's yard in relation to a management practice | 9 | 16 |
| Predator | Chickens being predated, or concerns of chickens being lost to predators | 14 | 34 |
| Protect | Protecting chickens | 13 | 22 |
| Separate | Separating animals from humans and the home/things in the home with respect to management practices | 18 | 58 |
| Smell | The smell of chickens with respect to management | 10 | 15 |
| Theft | Protecting chickens from theft/concerns about theft | 3 | 5 |
| Modifiers | Things that modify child/chicken relationships | 18 | 222 |
| Livestock | Productive animals not including chickens | 18 | 71 |
| Modern | Mentioning living a modern lifestyle | 2 | 3 |
| Money | Money/finance/assets or lack thereof | 14 | 42 |
| Program | An individual from a program/govt/NGO/health worker | 10 | 19 |
| Season | Seasonality in agricultural production, human or animal health, income fluctuation | 11 | 27 |
| Time | Time allocation/use, lack of time | 17 | 60 |
| Nutrition | General mention of nutrition | 18 | 198 |
| Child nut | The child’s nutrition | 17 | 95 |
| Growth | The role of nutrition in child growth | 12 | 17 |
| Diverse | Diversity of diet | 9 | 13 |
| Eggs | If they mention eggs for child nutrition | 16 | 44 |
| Fasting | Fasting of child/others | 2 | 4 |
| Knowledge | Having/not having knowledge of nutrition or health, or desire to expand knowledge of nutrition/health, or lack of knowledge as a barrier | 5 | 5 |
| Meat | Meat for child nutrition | 10 | 15 |
| WASH | Water, sanitation and hygiene | 18 | 283 |
| Cleaning | Cleaning up in general, sweeping up the yard, etc. | 15 | 43 |
| Flies | Flies as an aspect of cleanliness or WASH | 9 | 18 |
| Food | Any mention of food with respect to being clean/unclean/contaminated | 11 | 33 |
| Health | The health aspects/consequences of WASH | 16 | 68 |
| Interact | Humans and chickens interacting/playing/touching seen as unclean | 18 | 31 |
| Poop | Any reference to chicken poop/feces w.r.t. cleanliness | 17 | 44 |
| Waste | Role of waste in cleanliness | 12 | 27 |

^i^Child codes are indented under higher level parent codes. The columns are as follows: “Name” is the name of the code; “description” refers to the definition of the code used for coding purposes; “Files” refers to the number of separate transcripts (out of 18) in which the code was mentioned; “References” refers to the total number of times that the code was mentioned throughout all text.
